# Synthesis and Biological Profiling of New 1,2,3,4-Tetrahydrobenzo[*h*]naphthyridine-Based Hybrids as Dual Inhibitors of β-Amyloid and Tau Aggregation with Anticholinesterase Activity

**DOI:** 10.3390/biom16040593

**Published:** 2026-04-16

**Authors:** Aldrick B. Verano, Anna Sampietro, Ana Mallo-Abreu, Rosaria Spagnuolo, Belén Pérez, Manuela Bartolini, María Isabel Loza, José Brea, Jordi Juárez-Jiménez, Raimon Sabate, Carles Galdeano, Diego Muñoz-Torrero

**Affiliations:** 1 Laboratory of Medicinal Chemistry, Faculty of Pharmacy and Food Sciences, University of Barcelona, Av. Joan XXIII 27-31, E-08028 Barcelona, Spain; aldrickverano@gmail.com (A.B.V.); annasampietro@ub.edu (A.S.); ana.mallo.abreu@ub.edu (A.M.-A.); 2 Institute of Biomedicine of the University of Barcelona (IBUB), Av. Diagonal 643, E-08028 Barcelona, Spain; rsabate@ub.edu (R.S.); cgaldeano@ub.edu (C.G.); 3 Department of Pharmacy and Biotechnology, University of Bologna, Via Belmeloro 6, I-40126 Bologna, Italy; rosaria.spagnuolo2@unibo.it (R.S.); manuela.bartolini3@unibo.it (M.B.); 4 Department of Pharmacology, Therapeutics and Toxicology, Autonomous University of Barcelona, E-08193 Bellaterra, Spain; belen.perez@uab.cat; 5 BioFarma Research Group, Centro Singular de Investigación en Medicina Molecular y Enfermedades Crónicas (CIMUS), Departamento de Farmacología, Farmacia y Tecnología Farmacéutica, Universidade de Santiago de Compostela, Av. de Barcelona s/n, E-15782 Santiago de Compostela, Spain; mabel.loza@usc.es (M.I.L.); pepo.brea@usc.es (J.B.); 6 Department of Pharmacy and Pharmaceutical Technology and Physical-Chemistry, Faculty of Pharmacy and Food Sciences, University of Barcelona, Av. Joan XXIII 27-31, E-08028 Barcelona, Spain; jordi.juarez@ub.edu; 7 Institut de Química Teòrica i Computacional (IQTC), Facultat de Química i Física, Universitat de Barcelona, C. Martí i Franqués 1, E-08028 Barcelona, Spain

**Keywords:** multitarget agents, Alzheimer’s disease, amyloid aggregation inhibition, acetylcholinesterase inhibition, DMPK properties

## Abstract

DP-128 is a multitarget benzonaphthyridine-6-chlorotacrine hybrid molecule with potent in vitro anticholinesterase and Aβ42 and tau anti-aggregating activity. While often used as a reference protein aggregation inhibitor, its further development as an anti-Alzheimer agent is limited by significant cytotoxicity, suboptimal aqueous solubility and microsomal stability. Since these drawbacks might arise from its rather high lipophilicity, in this work we have developed a series of more polar analogues, designed by structural modifications at the benzonaphthyridine or 6-chlorotacrine moieties or within the eight-atom linker. Half of the new analogues are indeed slightly more soluble and clearly less cytotoxic than DP-128, display single-digit acetylcholinesterase inhibitory activity, and retain the Aβ42 and tau anti-aggregating potency of the lead, as well as favourable brain permeation and high plasma stability. While further optimization of microsomal stability is necessary for a potential therapeutic use of this class of compounds, hybrids **16** and **17**, with similar or even higher Aβ42 and tau anti-aggregating activity and lower cytotoxicity than DP-128, might represent novel pharmacological tools for protein aggregation studies.

## 1. Introduction

Dementia is one of the most worrisome leading causes of death worldwide, a major cause of disability and dependency in the elderly, and a huge economic burden for national economies. Accounting for most cases of dementia, Alzheimer’s disease (AD) is the main contributor to this health and socioeconomic impact, which, worryingly, is at a continuous rise along with population ageing, one of its main risk factors. The devastating effects of AD have been ascribed to the lack of drugs that are able to stop or slow down the progression of neurodegeneration by addressing the main pathogenic factors, which have remained elusive until recently. Indeed, after decades of intensive research with limited therapeutic breakthroughs in AD, the past five years have witnessed an important turning point in the field. Notably, the first three monoclonal antibodies (aducanumab [1], lecanemab [2], and donanemab [3,4]) targeting the β-amyloid peptide (Aβ), one of the main pathological hallmarks of AD [5], have received regulatory approval in the period 2021–2025. These drugs were designed to promote the clearance of aggregated Aβ species from the brain, thereby directly addressing amyloid pathology as a disease-modifying strategy. Although aducanumab was withdrawn from the market shortly after its controversial approval [6], lecanemab and donanemab have demonstrated the ability to modestly slow the clinical progression of AD, with reported reductions in cognitive decline in the range of approximately 25–30%, without, however, halting the underlying neurodegenerative process. These outcomes suggest that amyloid-β accumulation, while central to AD pathology, is unlikely to be the only driver of disease progression, and that additional pathogenic mechanisms contribute to neurodegeneration [7]. Indeed, in the last 15 years increasing attention has been paid to the multifactorial essence of neurodegenerative diseases and the logical seek of treatments that address more than one pathogenic factor, either in the form of combination therapies or single-molecule multitarget agents [8,9,10,11,12,13,14]. In line with this, (i) the diagnostic tools developed for the early detection of AD not only address cerebrospinal fluid (CSF) or blood Aβ levels or Aβ plaque but also the CSF or blood levels of tau protein, another key actor in AD neurodegeneration [15,16]; (ii) Aβ and tau are among the most pursued targets in the AD pipeline trials, being targeted by 18% and 11%, respectively, of all drug candidates undergoing clinical trials for AD in 2025 [17]; and (iii) drug combinations and multitarget drug candidates are also represented in the 2025 AD pipeline, with 10 drug combinations (5% of all trials) and 3 multitarget candidates (2% of all therapies) being tested [17].

**Figure 1:**
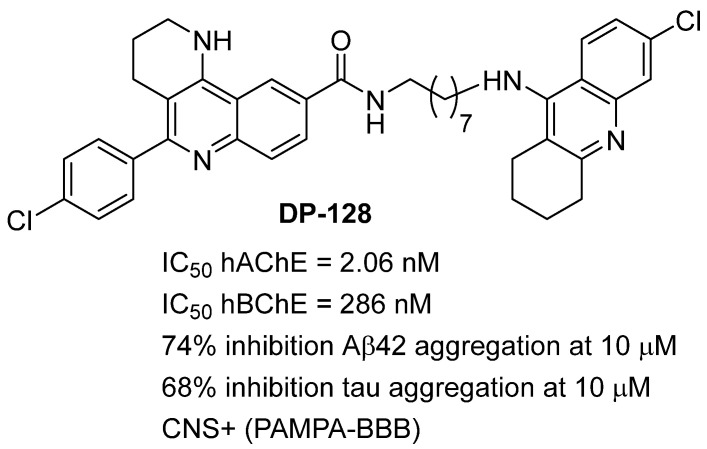
Chemical structure and biological profile of DP-128.

Apart from the Aβ-directed monoclonal antibodies, three out of the other four drugs approved for AD treatment are cholinesterase inhibitors (donepezil, galantamine, rivastigmine) [18]. They are purposed to decrease the metabolic hydrolysis of the neurotransmitter acetylcholine (ACh) by the enzymes acetylcholinesterase (AChE) and/or butyrylcholinesterase (BChE), thereby increasing central nervous system (CNS) ACh levels and coping with the decreased cholinergic neurotransmission that is behind the cognitive impairment in AD patients [19,20]. These drugs do not significantly alter Aβ or tau pathologies and are regarded as symptomatic. However, it should be noted that they provide sustained cognitive benefits [21], may slow the progression to severe cognitive and functional impairment [22], decrease mortality [23], reduce neuropsychiatric symptoms [24], and, in combination with the glutamate NMDA receptor antagonist memantine, their use leads to reduced burden and improved quality of life of AD patients and caregivers [25]. In contrast to disease-modifying drugs, like the Aβ-directed monoclonal antibodies or tau-targeting drug candidates in clinical trials, for which most benefit would be achieved by initiating treatment as soon as possible, the use of symptomatic cholinesterase inhibitors does not depend so critically on the disease stage upon treatment initiation. This has led to the suggestion that the concomitant use of disease-modifying and symptomatic drugs should lead to additive effects, thereby justifying the combination of both types of drugs or mechanisms to get a maximal benefit-to-risk ratio [26]. In this light, concomitant mitigation of Aβ and tau pathologies and inhibition of cholinesterases seems a sound approach to reach the aforementioned additive effects. Some years ago, we developed a new class of benzo[*h*][1,6]naphthyridine-6-chlorotacrine hybrids having such a multitarget profile. The lead of this family, DP-128 (Figure 1), is a potent inhibitor of both Aβ42 and tau aggregation. Indeed, DP-128 at a 10 µM concentration inhibits Aβ42 and tau aggregation by 74% and 68%, respectively, in live *Escherichia coli* cells that overexpress one or the other protein [27]. We have even demonstrated that DP-128 is a pan-amyloid inhibitor that is able to block the aggregation of a variety of amyloidogenic proteins involved in AD and other neurological disorders, in non-neurological disorders, and also inhibits the aggregation of bacterial and fungal proteins [28]. The antiaggregative properties of DP-128 are accompanied by a potent in vitro inhibition of human AChE activity (IC_50_ = 2.06 nM) and human BChE (IC_50_ = 286 nM) and excellent permeability across an in vitro model of blood–brain barrier (BBB) [27] (Figure 1).

To further assess its potential as a disease-modifying anti-AD drug candidate, we have determined its cytotoxicity and some drug metabolism and pharmacokinetics (DMPK) properties experimentally. We have found that DP-128 has high plasma stability, with the compound remaining essentially unchanged after 1 h incubation in human, mouse, and rat plasma. However, the aqueous solubility of DP-128 is only moderate (14.7 µM) and its microsomal stability is very poor, with only 1.5, 2.7, and 35.7% compound remaining non-metabolized after 1 h incubation with human, mouse, and rat microsomes at 37 °C, but its most concerning limitation is a significant cytotoxicity, with a LD_50_ < 10 µM in neuroblastoma SH-SY5Y cells. Prompted by the outstanding multitarget profile of DP-128, we envisaged a lead optimization campaign to afford novel analogues that might retain or even improve the potencies against Aβ42 and tau aggregation and cholinesterase inhibition of the lead DP-128 and overcome its physicochemical, DMPK, and cytotoxicity limitations.

Lipophilicity is generally inversely related to aqueous solubility. Also, high lipophilicity is usually associated with rapid metabolic clearance and cytotoxicity [29,30]. In turn, high molecular weight seems to be associated with poor physicochemical properties [31,32,33]. Actually, DP-128 is a quite lipophilic molecule (log P = 9.3, predicted with Molinspiration [34]; log P = 8.5, predicted with SwissADME [35] with a relatively high molecular weight (680.7). Since these properties might be behind the poor aqueous solubility, high microsomal turnover, and significant cytotoxicity of DP-128, we have designed new analogues by structural modifications directed to increase polarity/reduce lipophilicity and reduce molecular weight to overcome the physicochemical, DMPK, and toxicity limitations of the lead without losing potency at its different biological targets.

## 2. Materials and Methods

### 2.1. Synthesis of the Target Compounds: General Methods

Reaction progress was monitored with silica gel 60 F254 TLC plates (ref. 1.05554, EMD Millipore, Merck, Darmstadt, Germany) and visualized with 254 nm and 365 nm ultraviolet light and/or with 1% aqueous solution of KMnO_4_ and then mildly charred by a heat gun. Column chromatography was performed on silica gel 60 AC.C (35–70 mesh, ref 11438812, Fisher Chemicals, Germany). Melting points were determined in open capillary tubes using an MFB 59510M Gallenkamp apparatus (Loughborough, UK) and are uncorrected. A Perkin-Elmer Spectrum RX I spectrophotometer (Waltham, MA, USA) was used to acquire the IR spectra. Only significant absorption bands, expressed as wavenumbers (cm^−1^), are given. ^1^H NMR (400 MHz) and ^13^C NMR (100.6 MHz) spectra were recorded at the *Unitat d’RMN* of the Centres Científics i Tecnològics of the Universitat de Barcelona (CCiTUB) using a Bruker Avance NEO spectrometer (Wissembourg, France). NMR spectra are described by chemical shifts (δ values, in ppm) referenced to solvent signals and coupling constants (*J* values, in Hz). High-resolution mass spectrometry (HRMS) was performed with an Agilent Technologies LC/MSD-TOF mass spectrometer (Santa Clara, CA, USA) at the *Unitat d’Espectrometria de Masses de Caracterització Molecular* of the CCiTUB. Analytical HPLC was used to assess the purity of the newly synthesized compounds with the following equipment and procedure: equipment: Agilent 1260 Infinity II LC/MSD system (Santa Clara, CA, USA) coupled to a photodiode array detector and a mass spectrometer; sample preparation: five mL samples at 0.5 mg/mL in 0.05% formic acid in water (A) and 0.05% formic acid in acetonitrile (B) 1:1 mixtures; column: Agilent Poroshell 120 EC-C18 (2.7 μm, 50 mm × 4.6 mm, Santa Clara, CA, USA), thermostated at 40 °C; mobile phase and conditions: mixtures of solutions A and B, flow rate of 0.6 mL/min, gradient from 95% A − 5% B to 100% B in 3 min, 100% B for 3 min, from 100% B to 95% A − 5% B in 1 min, and 95% A − 5% B for 3 min. Purity was calculated as the percentage of the total peak area at 254 nm. All samples of the new compounds subjected to pharmacological evaluation exhibited a purity of >95%.

#### 2.1.1. Ethyl 1-(*tert*-butoxycarbonyl)-5-(thiazol-2-yl)-3,4-dihydrobenzo[*h*][1,6]naphthyridine-9-carboxylate (**6**)

To a solution of thiazole-2-carbaldehyde (0.5 mL, 5.52 mmol) and ethyl 4-aminobenzoate (901 mg, 5.45 mmol) in dry acetonitrile (ACN, 21 mL), Yb(OTf)_3_ (677 mg, 1.09 mmol) was added. After stirring this mixture at room temperature for 5 min, *N*-Boc-3,4-dihydro-2*H*-pyridine (1.0 mL, 5.57 mmol) was added and the reaction mixture was stirred at room temperature for 3 days, and then diluted with sat. aq. NaHCO_3_ (40 mL), and extracted with EtOAc (3 × 40 mL). The combined organic extracts were dried over anhydrous Na_2_SO_4_, filtered and evaporated under reduced pressure to give a deep blue-green crude oil (2.88 g). The crude product was taken up in dichloromethane (DCM, 8 mL) and treated with 2,3-dichloro-5,6-dicyano-1,4-benzoquinone (DDQ, 2.95 g, 13.0 mmol) with stirring at room temperature overnight. The mixture was diluted with sat. aq. NaHCO_3_ (50 mL). The aqueous phase was extracted with DCM (4 × 30 mL) and the combined organic layers were washed with a saturated aqueous solution of NaHCO_3_ (2 × 100 mL), dried over anhydrous Na_2_SO_4_, and evaporated under vacuum. Chromatographic purification of the crude on silica gel, eluting with hexane/EtOAc 80:20, provided compound **6** (388 mg, 16% overall yield) as white solid; *R*_f_ = 0.46 (hexane/EtOAc 3:2); IR (ATR-FTIR) ν_max_ (cm^−1^): 3413, 3132, 2580, 2861, 2795, 1687, 1420, 1091, 844; ^1^H NMR (400 MHz, CDCl_3_) δ (ppm): 8.58 (d, *J* = 2.0 Hz, 1H, 10-H), 8.23 (dd, *J* = 8.8 Hz, *J’* = 2.0 Hz, 1H, 8-H), 8.07 (d, *J* = 8.8 Hz, 1H, 7-H), 7.99 (d, *J* = 3.2 Hz, 1H, thiazole 4-H), 7.51 (d, *J* = 3.2 Hz, 1H, thiazole 5-H), 4.44 (q, *J* = 7.2 Hz, 2H, 9-CO_2_C*H*_2_CH_3_), 3.81–3.02 (m, 4H, 2-H_2_, 4-H_2_), 2.12 (m, 2H, 3-H_2_), 1.43 (t, *J* = 7.2 Hz, 3H, 9-CO_2_CH_2_C*H*_3_), 1.32 [br s, 9H, OC(CH_3_)_3_]; ^13^C NMR (101 MHz, CDCl_3_) δ (ppm): 170.7 [C, thiazole C2], 166.4 (C, 9-*C*OOCH_2_CH_3_), 154.0 (C, NCOO), 151.3 (C, C5), 148.3 (C, C6a), 147.0 (C, C10b), 144.1 (CH, thiazole C4), 129.7 (CH, C7), 128.5 (CH, C8), 127.8 (CH, C10), 127.6 (C, C9), 123.9 (CH, thiazole C5), 123.7 (C, C10a), 122.7 (C, C4a), 82.2 [C, O*C*(CH_3_)_3_], 61.5 (CH_2_, 9-COO*C*H_2_CH_3_), 44.7 (CH_2_, C2), 28.0 [3CH_3_, OC(*C*H_3_)_3_], 25.1(CH_2_, C4), 23.6 (CH_2_, C3), 14.5 (CH_3_, 9-COOCH_2_*C*H_3_); HRMS (ESI-TOF) *m*/*z* calculated for [C_23_H_25_N_3_O_4_S + H]^+^: 440.1639, found 440.1644.

#### 2.1.2. Ethyl 1-(*tert*-butoxycarbonyl)-5-(pyridin-3-yl)-3,4-dihydrobenzo[*h*][1,6]naphthyridine-9-carboxylate (**7**)

This compound was synthesized using the same conditions described for compound **6**. From a solution of pyridine-3-carbaldehyde (0.6 mL, 6.27 mmol) and ethyl 4-aminobenzoate (992 mg, 6.00 mmol) in dry ACN (23 mL), Yb(OTf)_3_ (745 mg, 1.20 mmol), and *N*-Boc-3,4-dihydro-2*H*-pyridine (1.0 mL, 5.57 mmol), a crude yellow oil (4.16 g) was obtained. A portion (3.00 g) of this crude was taken up in DCM (10 mL) and oxidized with DDQ (3.11 g, 13.7 mmol). After silica gel chromatographic purification of the reaction crude, using hexane/EtOAc mixtures 80:20 to 30:70, compound **7** (1.80 g, 75% overall yield) was obtained as a brown solid [36]; ^1^H NMR (400 MHz, CDCl_3_) δ (ppm): 8.87 (d, *J* = 2.0 Hz, 1H, pyridine 2-H), 8.71 (dd, *J* = 4.8 Hz, *J’* = 2.0 Hz, 1H, pyridine 6-H), 8.60 (d, *J* = 2.0 Hz, 1H, 10-H), 8.25 (dd, *J* = 8.8 Hz, *J* = 2.0 Hz, 1H, 8-H), 8.09 (d, *J* = 8.8 Hz, 1H, 7-H), 7.96 (ddd, *J* = 7.6 Hz, *J’* = *J”* = 2.0 Hz, 1H, pyridine 4-H), 7.46 (ddd, *J* = 7.6 Hz, *J’* = 4.8 Hz, *J”* = 0.4 Hz, 1H, pyridine 5-H), 4.45 (q, *J* = 7.0 Hz, 2H, 9-CO_2_C*H*_2_CH_3_), 2.83 (t, *J* = 6.4 Hz, 1H, 2-H_2_), 2.14–1.89 (m, 4H, 3-H_2_, 4-H_2_), 1.49–1.28 [m, 12H, 9-CO_2_CH_2_C*H*_3_, OC(C*H*_3_)_3_].

#### 2.1.3. Ethyl 1-(*tert*-butoxycarbonyl)-5-(pyridin-4-yl)-3,4-dihydrobenzo[*h*][1,6]naphthyridine-9-carboxylate (**8**)

This compound was synthesized using the same conditions described for compound **6**. From a solution of pyridine-4-carbaldehyde (0.6 mL, 6.27 mmol) and ethyl 4-aminobenzoate (988 mg, 5.98 mmol) in dry ACN (25 mL), Yb(OTf)_3_ (742 mg, 1.20 mmol), and *N*-Boc-3,4-dihydro-2*H*-pyridine (1.0 mL, 5.57 mmol), a crude yellow oil (3.48 g) was obtained. A portion (2.88 g) of this crude was taken up in DCM (10 mL) and oxidized with DDQ (4.32 g, 19.0 mmol). After silica gel chromatographic purification of the reaction crude, using hexane/EtOAc mixtures 80:20 to 30:70, compound **8** (188 mg, 8% overall yield) was obtained as a brown solid; *R*_f_ = 0.52 (hexane/EtOAc 1:1); IR (ATR-FTIR) ν_max_ (cm^−1^): 2995, 2865, 1695, 1643, 1591, 1285, 1241; ^1^H NMR (400 MHz, CDCl_3_) δ (ppm): 8.77–8.60 [m, 2H, pyridine 2(6)-H], 8.60 (d, *J* = 2.0 Hz, 1H, 10-H), 8.25 (dd, *J* = 8.8 Hz, *J’* = 2.0 Hz, 1H, 8-H), 8.09 (d, *J* = 8.8 Hz, 1H, 7-H), 7.52– 7.51 [m, 2H, pyridine 3(5)-H], 4.45 (q, *J* = 7.2 Hz, 2H, 9-CO_2_C*H*_2_CH_3_), 2.80 (t, *J* = 6.4 Hz, 2H, 2-H_2_), 2.15–1.72 (m, 4H, 3-H_2_, 4-H_2_), 1.45–1.35 [m, 12H, 9-CO_2_CH_2_C*H*_3_, OC(C*H*_3_)_3_]; ^13^C NMR (101 MHz, CDCl_3_) δ (ppm): 166.4 (C, 9-*C*O_2_CH_2_CH_3_), 159.1 (C, C5), 154.0 (C, NCOO), 150.1 [2CH, pyridine C2(6)], 149.0 (C, C6a), 147.7 (C, C10b), 131.0 (C, pyridine C4), 130.0 (CH, C7), 128.6 (CH, C8), 127.8 (CH, C10), 127.6 (C, C9), 123.7 [2CH, pyridine C3(5)], 123.4 (C, C4a), 119.6 (C, C10a), 82.4 [C, O*C*(CH_3_)_3_], 61.5 (CH_2_, 9-COO*C*H_2_CH_3_), 44.9 (CH_2_, C2), 28.1 [3CH_3_, OC(*C*H_3_)_3_], 25.6 (CH_2_, C4), 24.1 (CH_2_, C3), 14.5 (CH_3_, 9-COOCH_2_*C*H_3_); HRMS (ESI-TOF) *m*/*z* calculated for [C_25_H_27_N_3_O_4_ + H]^+^: 434.2074, found 434.2074.

#### 2.1.4. 5-(Thiazol-2-yl)-3,4-dihydrobenzo[*h*][1,6]naphthyridine-9-carboxylic acid (**9**)

A solution of ester **6** (150 mg, 0.34 mmol) in MeOH (2 mL) was treated dropwise with 40% aq. KOH (1.3 mL) and the mixture was stirred under reflux overnight. The solvent was evaporated under reduced pressure and the resulting residue was taken up in MeOH (4 mL), treated dropwise with an HCl solution in dioxane (4M solution, 0.5 mL), and stirred at room temperature for 30 min. Finally, the solvent was evaporated to dryness, to afford crude carboxylic acid **9** in the form of the dihydrochloride salt (162 mg), which was used in the following step without further purification; IR (ATR-FTIR) ν_max_ (cm^−1^): 3608, 2950, 2664, 1795, 1673, 1591, 1285, 1207, 643; ^1^H NMR (400 MHz, CD_3_OD) δ (ppm): 9.24 (br s, 1H), superimposed 9.09 (d, *J* = 1.6 Hz, 1H, 10-H), 9.08 (br s, 1H), 8.78 (d, *J* = 7.6 Hz, 1H, thiazole 4-H), 8.46 (dd, *J* = 8.8 Hz, *J* = 1.6 Hz, 1H, 8-H), 8.21 (dd, *J* = 7.6 Hz, *J’* = 5.6 Hz, 1H, thiazole 5-H), 7.93 (d, *J* = 8.8 Hz, 1H, 7-H), 3.74 (t, *J* = 5.4 Hz, 1H, 2-H_2_), 2.78 (t, *J* = 6.0 Hz, 1H, 4-H_2_), 2.03 (m, 1H, 3-H_2_); ^13^C NMR (101 MHz, CD_3_OD) δ (ppm): 167.7 (C, 9-CO_2_H), 156.3 (C, C10b), 147.2 (C, C5), 146.0 (C, thiazole C2), 145.7 (CH, thiazole C4), 141.3 (C, C6a), 134.7 (C, C9), 130.5 (CH, C8), 128.1 (CH, thiazole C5), 126.4 (CH, C10), 121.2 (CH, C7), 116.4 (C, C10a), 111.0 (C, C4a), 43.2 (CH_2_, C2), 24.6 (CH_2_, C4), 19.8 (CH_2_, C3); HRMS (ESI-TOF) *m*/*z* calculated for [C_16_H_12_N_3_O_2_S + H]^+^: 312.0801, found 312.0806.

#### 2.1.5. 5-(Pyridin-3-yl)-3,4-dihydrobenzo[*h*][1,6]naphthyridine-9-carboxylic acid (**10**)

This compound was synthesized using the same conditions described for compound **9**. From a solution of ester **7** (110 mg, 0.25 mmol) in MeOH (1.5 mL) and 40% aq. KOH (1.8 mL), a residue was obtained and taken up in MeOH (3.5 mL) and treated with an HCl solution in dioxane (4M solution, 0.5 mL) to finally afford the carboxylic acid **10** in the form of the dihydrochloride salt (124 mg), which was used in the following step without further purification [36]: ^1^H NMR (400 MHz, CD_3_OD) *δ* (ppm): 8.70 (dd, *J =* 2.0 Hz, *J’* = 0.4 Hz, 1H, 10-H), 8.62 (dd, *J* = 4.8 Hz, *J’* = 2.0 Hz, 1H, pyridine 6-H), 8.56 (dd, *J* = 1.6 Hz, *J’* = 0.8 Hz, 1H, pyridine 2-H), 8.16 (dd, *J =* 8.8 Hz, *J’* = 2.0 Hz, 1H, 8-H), 8.01 (ddd, *J* = 8.0 Hz, *J’* = 2.0 Hz, *J”* = 1.6 Hz, 1H, pyridine 4-H), 7.76 (dd, *J* = 8.8 Hz, *J’* = 0.4 Hz, 1H, 7-H), 7.57 (ddd, *J* = 8.0 Hz, *J’* = 4.8 Hz, *J”* = 0.8 Hz, 1H, pyridine 5-H), 3.56 (t, *J* = 6.0 Hz, 1H, 2-H_2_), 2.69 (t, *J* = 6.0 Hz, 1H, 4-H_2_), 1.91 (tt, *J* = *J’* = 6.0 Hz, 1H, 3-H_2_).

#### 2.1.6. 5-(Pyridin-4-yl)-3,4-dihydrobenzo[*h*][1,6]naphthyridine-9-carboxylic acid (**11**)

This compound was synthesized using the same conditions described for compound **9**. From a solution of ester **8** (100 mg, 0.23 mmol) in MeOH (1.5 mL) and 40% aq. KOH (1.8 mL), a residue was obtained and taken up in MeOH (3.5 mL) and treated with an HCl solution in dioxane (4M solution, 0.5 mL) to finally afford the carboxylic acid **11** in the form of the dihydrochloride salt (115 mg), which was used in the following step without further purification: IR (ATR-FTIR) *ν*_max_ (cm^−1^): 3558, 2751, 2774, 1795, 1621, 1540, 1205, 1209, 651; ^1^H NMR (400 MHz, CD_3_OD) *δ* (ppm): 9.07 (br s, 1H, 10-H), 8.87 [d, *J* = 5.6 Hz, 2H, pyridine 2(6)-H], 8.45 (br d, *J* = 8.4 Hz, 1H, 8-H), 7.89 (d, *J* = 8.4 Hz, 1H, 7-H), 7.74 [m, 2H, pyridine 3(5)-H], 3.73 (t, *J* = 6.0 Hz, 2H, 2-H_2_), 2.75 (t, *J* = 6.0 Hz, 1H, 4-H_2_), 2.01 (tt, *J* = *J’* = 6.0 Hz, 1H, 3-H_2_); HRMS (ESI-TOF) *m/z* calculated for [C_18_H_15_N_3_O_2_ + H]^+^: 306.1237, found 306.1244.

#### 2.1.7. *N*-(6-Chloro-1,2,3,4-tetrahydroacridin-9-yl)octane-1,8-diamine (**14**)

A mixture of chloroquinoline **12** (1.00 g, 3.97 mmol), 1,8-octanediamine (2.86 g, 19.8 mmol), KI (33 mg, 0.20 mmol) and phenol (373 mg) was stirred at 180 °C for 3 h, and then cooled to 40 °C, alkalinized with 1N NaOH (7 mL), and extracted with DCM (3 × 10 mL). The combined organic layers were washed with brine (3 × 10 mL), dried over anhydrous Na_2_SO_4,_ filtered, and concentrated under reduced pressure. Purification of the crude was carried out using pre-packed silica gel cartridges-RediSep^®^- onto Isco-CombiFlash^®^ Companion^®^ (DCM/Ammonia, ca. 7N solution in MeOH 10:0 to 9:1). The amine **14** (278 mg, 19% yield) was obtained as a light-yellow oil [37]; *R*_f_ = 0.51 (DCM/ammonia, ca. 7N solution in MeOH 9:1); ^1^H NMR (400 MHz, CD_3_OD) *δ* (ppm): 8.10 (d, *J* = 9.2 Hz, 1H, 8′-H), 7.74 (d, *J* = 2.0 Hz, 1H, 5′-H), 7.32 (dd, *J* = 9.2 Hz, *J’* = 2.0 Hz, 1H, 7′-H), 3.55 (t, *J* = 7.2 Hz, 2H, 1-H_2_), 2.97 (t, *J* = 4.6 Hz, 2H, 4′-H_2_), 2.73 (t, *J* = 4.6 Hz, 2H, 1′-H_2_), 2.59 (t, *J* = 7.2 Hz, 2H, 8-H_2_), 1.91 (m, 4H, 2′ -H_2_, 3′-H_2_), 1.64 (tt, *J* = *J’* = 7.2 Hz, 2H, 2-H_2_), 1.42 (tt, *J* = *J’* = 7.2 Hz, 2H, 7-H_2_), 1.36–1.25 (m, 8H, 3-H_2_, 4-H_2_, 5-H_2_, 6-H_2_).

#### 2.1.8. *N*-{8-[(6-Chloro-1,2,3,4-tetrahydroacridin-9-yl)amino]octyl}-5-(thiazol-2-yl)-1,2,3,4-tetrahydrobenzo[*h*][1,6]naphthyridine-9-carboxamide (**15**)

A solution of amine **14** (123 mg, 0.34 mmol) and carboxylic acid **9** (319 mg of crude product, 1.03 mmol) in dry DMF (2.5 mL) was treated dropwise with diisopropylethylamine (DIPEA, 0.1 mL, 0.57 mmol) and the mixture was stirred at room temperature for 10 min. Then, a solution of hexafluorophosphate azabenzotriazole tetramethyl uronium (HATU, 200 mg, 0.53 mmol) in dry dimethylformamide (DMF, 1.5 mL) was added and the reaction mixture was stirred at room temperature overnight. The solvent was evaporated to dryness and the residue was purified by silica gel column chromatography, eluting with DCM/ammonia, ca. 7N solution in MeOH 90:10. The desired hybrid **15** (13 mg, 6% yield) was obtained as a brown solid; *R*_f_ = 0.32 (DCM/ammonia, ca. 7N solution in MeOH 9:1).

For the biological profiling, hybrid **15** was converted into the corresponding trihydrochloride salt as follows: a solution of **15** (13 mg) in DCM (2 mL) was treated with 5M HCl in isopropanol (1 mL). The solvent was evaporated to dryness and the solid was washed with pentane (3 × 2 mL), to afford **15**·3HCl (15 mg) as a brown solid; mp: 197–199 °C; IR (ATR-FTIR) *ν*_max_ (cm^−1^): 3339, 3193, 3107, 3080, 1715, 1627, 1579, 1324, 775; ^1^H NMR (400 MHz, CD_3_OD) *δ* (ppm): 8.92 (br s, 1H, 10-H), 8.65 (m, 1H, NH), 8.36 (br d, *J* = 9.2 Hz, 1H, 8-H), 8.27 (d, *J* = 3.2 Hz, 1H, thiazole 4-H), superimposed in part 8.26 (m, 1H, 8”-H), 8.19 (d, *J* = 3.2 Hz, 1H, thiazole 5-H), 8.02 (d, *J* = 9.2 Hz, 1H, 7-H), 7.75 (br s, 1H, 5”-H), 7.53 (br d, *J* = 9.2 Hz, 1H, 7”-H), 3.93 (t, *J* = 7.2 Hz, 2H, 8′-H_2_), 3.74 (t, *J* = 4.8 Hz, 2H, 2-H_2_), 3.45 (t, *J* = 6.4 Hz, 1′-H_2_), 3.14 (t, *J* = 4.8 Hz, 2H, 4-H_2_), 2.98 (t, *J* = 4.6 Hz, 2H, 4”-H_2_), 2.61 (t, *J* = 5.4 Hz, 2H, 1”-H_2_), 2.12 (m, 2H, 3-H_2_), 1.97–1.93 (m, 4H, 2”-H_2_, 3”-H_2_), 1.84 (tt, *J* = *J’* = 6.8 Hz, 2H, 7′-H_2_), 1.69 (tt, *J* = *J’* = 6.8 Hz, 2H, 2′-H_2_), 1.50–1.41 (m, 8H, 3′-H_2_, 4′-H_2_, 5′-H_2_, 6′-H_2_); ^13^C NMR (101 MHz, CD_3_OD) *δ* (ppm): 171.4 (C, 1-CONH), 169.7 (C, thiazole C-2), 159.8 (C, C9”), 153.6 (C, C10b), 151.6 (C, C4a”), 150.8 (C, C5), 149.1 (C, C10a”), 148.1 (C, C6a), 144.2 (C, C6”), 135.7 (C, C9), 131.6 (CH, C8), 129.7 (CH, C8”), 126.6 (CH, C7”), 126.2 (CH, C10), 125.1 (CH, C7), 123.1 (CH, thiazole C-4), 122.2 (CH, C5”), 119.2 (C, C10a), 118.1 (CH, C8a”), 116.6 (C, C9a”), 116.5 (CH, thiazole C-5), 109.5 (C, C4a), 49.8 (CH_2_, C8′), 42.1 (CH_2_, C2), 41.1 (CH_2_, C1′), 34.0 (CH_2_, C7′), 32.1 (CH_2_, C2′), 30.4 (CH_2_), 30.2 (2CH_2_) (C4′, C5′, C4”), 27.8 (CH_2_, C3′), 27.7 (CH_2_, C6′), 25.88 (CH_2_, C4), 25.86 (CH_2_, C1”), 23.9 (CH_2_, C2”), 23.5 (CH_2_, C3), 21.7 (CH_2_, C3”); HRMS (ESI-TOF) *m/z* calculated for [C_37_H_42_^35^ClN_6_OS + H]^+^ 653.2820, found 653.2824.

#### 2.1.9. *N*-{8-[(6-Chloro-1,2,3,4-tetrahydroacridin-9-yl)amino]octyl}-5-(pyridin-3-yl)-1,2,3,4-tetrahydrobenzo[*h*][1,6]naphthyridine-9-carboxamide (**16**)

This compound was synthesized using the same conditions described for hybrid **15**. From a solution of amine **14** (80 mg, 0.22 mmol) and carboxylic acid **10** (270 mg of crude product, 0.66 mmol) in dry DMF (2.5 mL), DIPEA (0.1 mL, 0.57 mmol), and a solution of HATU (127 mg, 0.33 mmol) in DMF (1.5 mL), hybrid **16** (13 mg, 9% yield) was obtained, as a yellow solid, after silica gel column chromatography purification, eluting with DCM/ammonia, ca. 7N solution in MeOH 90:10; *R*_f_ = 0.48 (DCM/ammonia, ca. 7N solution in MeOH 9:1).

The analytical sample of **16**·3HCl was prepared as described before, upon treatment with 5M HCl in isopropanol (1 mL), washing of the solid with pentane (3 × 2 mL) and drying in vacuo; mp 202–203 °C; IR (ATR-FTIR) *ν*_max_ (cm^−1^): 3095, 2932, 2936, 1685, 1593, 1399, 1185, 1067, 782, 686; ^1^H NMR (400 MHz, CD_3_OD) *δ* (ppm): 8.70 (dd, *J* = 2.0 Hz, *J’* = 0.8 Hz, 1H, 10-H), 8.63 (dd, *J* = 4.8 Hz, *J’* = 1.6 Hz, 1H, pyridine 6-H), 8.52 (d, *J* = 2.0 Hz, 1H, pyridine 2-H), 8.05 (d, *J* = 9.2 Hz, 1H, 8”-H), 8.00 (ddd, *J* = 8.0 Hz, *J’* = 2.0 Hz, *J”* = 1.6 Hz, 1H, pyridine 4-H), 7.96 (dd, *J* = 8.8 Hz, *J’* = 2.0 Hz, 1H, 8-H), 7.81 (d, *J* = 8.8 Hz, 1H, 7-H), 7.71 (d, *J* = 2.0 Hz, 1H, 5”-H), 7.57 (ddd, *J* = 8.0 Hz, *J’* = 4.8 Hz, *J”* = 0.8 Hz, 1H, pyridine 5-H), 7.28 (dd, *J* = 9.2 Hz, *J’* = 2.0 Hz, 1H, 7”-H), 3.53 (m, 4H, 2-H_2_, 8′-H_2_), 3.41 (t, *J* = 2.2 Hz, 2H, 1′-H_2_), 2.94 (t, *J* = 6.4 Hz, 1H, 4”-H_2_), 2.70–2.67 (m, 4H, 4-H_2_, 1”-H_2,_), 1.93–1.86 (m, 6H, 3-H_2_, 2”-H_2_, 3”-H_2_), 1.65–1.61 (m, 4H, 2′-H_2_, 7′-H_2_), 1.41–1.31 (m, 8H, 3′-H_2_, 4′-H_2_, 5′-H_2_, 6′-H_2_); ^13^C NMR (101 MHz, CD_3_OD) *δ* (ppm): 169.7 (C, 1-CONH), 160.1 (C, C9”), 158.2 (C, C10b), 153.5 (C, C4a”), 150.5 (CH, pyridine C-6), 150.0 (C, pyridine C-2), 149.7 (C, C5), 149.1 (C, C10a”), 148.4 (C, C6a), 138.6 (C, C6”), 138.3 (C, pyridine C-4), 135.6 (C, C9), 131.5 (CH, C8), 129.0 (CH, C8”), 128.0 (CH, pyridine C5), 126.6 (C, pyridine C-3), 126.4 (CH, C7”), 125.0 (CH, C10), 124.9 (CH, C7), 122.4 (CH, C5”), 119.4 (C, C10a), 117.9 (C, C8a”), 116.6 (C, C9a”), 109.0 (C, C4a), 49.7 (CH_2_, C8′), 42.2 (CH_2_, C2), 41.1 (CH_2_, C1′), 34.1 (CH_2_, C7′), 32.2 (CH_2_, C2′), 30.4 (CH_2_), 30.2 (2CH_2_) (C4′, C5′, C4”), 27.8 (CH_2_, C3′), 27.7 (CH_2_, C6′), 26.3 (CH_2_, C4), 26.0 (CH_2_, C1”), 23.9 (CH_2_, C2”), 23.5 (CH_2_, C3”), 21.5 (CH_2_, C3); HRMS (ESI-TOF) *m/z* calculated for [C_39_H_44_^35^ClN_6_O + H]^+^ 647.3260, found 647.3259.

#### 2.1.10. *N*-{8-[(6-Chloro-1,2,3,4-tetrahydroacridin-9-yl)amino]octyl}-5-(pyridin-4-yl)-1,2,3,4-tetrahydrobenzo[*h*][1,6]naphthyridine-9-carboxamide (**17**)

This compound was synthesized using the same conditions described for hybrid **15**. From a solution of amine **14** (100 mg, 0.28 mmol) and carboxylic acid **11** (338 mg of crude product, 0.83 mmol) in dry DMF (2.5 mL), DIPEA (0.1 mL, 0.57 mmol), and a solution of HATU (158 mg, 0.42 mmol) in DMF (1.5 mL), hybrid **17** (22 mg, 12% yield) was obtained, as a yellow oil, after silica gel column chromatography purification, eluting with DCM/ammonia, ca. 7N solution in MeOH 90:10; *R*_f_ = 0.43 (DCM/ammonia, ca. 7N solution in MeOH 9:1).

The analytical sample of **17**·3HCl was prepared as described before, upon treatment with 5M HCl in isopropanol (1 mL), washing of the solid with pentane (3 × 2 mL) and drying in vacuo; mp 197–198 °C; IR (ATR-FTIR) ν_max_ (cm^−1^): 3237, 3093, 2924, 2851, 1625, 1573, 1322, 1089, 842; ^1^H NMR (400 MHz, CD_3_OD) δ (ppm): 9.14 [m, 2H, pyridine 2(6)-H], 9.00 (br s, 1H, 10-H), 8.39 (d, *J* = 8.8 Hz, 1H, 8”-H), 8.29 [d, *J* = 5.6 Hz, 2H, pyridine 3(5)-H], superimposed 8.29 (m, 1H, 8-H), 7.92 (d, *J* = 7.6 Hz, 1H, 7-H), 7.77 (br s, 1H, 5”-H), 7.55 (br d, *J* = 8.8 Hz, 1H, 7”-H), 3.94 (m, 2H, 8′-H_2_), 3.74 (m, 2H, 2-H_2_), 3.44 (t, *J* = 6.0 Hz, 2H, 1′-H_2_), 2.99 (m, 2H, 4”-H_2_), 2.76 (m, 2H, 4-H_2_), 2.68 (m, 2H, 1”-H_2_), 2.02–1.95 (m, 6H, 3-H_2_, 2”-H_2_, 3”-H_2_), 1.85 (s, 2H, 7′-H_2_), 1.68 (t, *J* = 7.2 Hz, 2H, 2′-H_2_), 1.46–1.42 (m, 8H, 3′-H_2_, 4′-H_2_, 5′-H_2_, 6′-H_2_); ^13^C NMR (101 MHz, CD_3_OD) δ (ppm): 167.9 (C, 9-CONH), 157.8 (C, C9”), 156.2 (C, C10b), 152.1 (C, C4a”), 148.7 (C, C5), 146.8 [C, pyridine C2(6)]), 140.5 (C, C10a”), 140.3 (C, C6a), 140.1 (C, C6”), 134.1 (C, pyridine C4), 133.1 (CH + C, C8, C9), 128.8 (CH, C8”), 126.8 [3CH, C7”, pyridine C3(5)]), 123.9 (CH, C10), 121.2 (CH, C7), 119.1 (CH, C5”), 116.4 (C, C10a), 115.4 (C, C8a”), 113.3 (C, C9a”), 110.3 (C, C4a), 49.7 (CH_2_, C8′), 43.2 (CH_2_, C2), 41.3 (CH_2_, C1′), 31.3 (CH_2_, C7′), 30.4 (CH_2_, C2′), 30.22 (CH_2_), 30.19 (CH_2_) (C4′, C5′), 29.4 (CH_2_, C4”), 28.0 (CH_2_, C3′), 27.7 (CH_2_, C6′), 24.8 (2CH_2_, C4, C1”), 22.9 (CH_2_, C2”), 21.8 (CH_2_, C3”), 19.9 (CH, C3); HRMS (ESI-TOF) *m*/*z* calculated for [C_39_H_44_^35^ClN_6_O + H]^+^ 647.3260, found 647.3261.

#### 2.1.11. Ethyl 1-(*tert*-butoxycarbonyl)-5-(4-chlorophenyl)-3,4-dihydrobenzo[*h*][1,6]naphthyridine-9-carboxylate (**19**)

This compound was synthesized using the same conditions described for compound **6**. From a solution of 4-chlorobenzaldehyde (805 mg, 5.73 mmol) and ethyl 4-aminobenzoate (947 mg, 5.73 mmol) in dry ACN (20 mL), Yb(OTf)_3_ (710 mg, 1.15 mmol), and *N*-Boc-3,4-dihydro-2*H*-pyridine (1.0 mL, 5.46 mmol), a crude light-yellow oil (3.83 g) was obtained. This crude was taken up in DCM (11 mL) and oxidized with DDQ (4.00 g, 17.6 mmol). After silica gel chromatographic purification of the reaction crude, using hexane/EtOAc 80:20, compound **19** (904 mg, 35% overall yield) was obtained as a yellow solid [36]; ^1^H NMR (400 MHz, CDCl_3_) δ (ppm): 8.59 (d, *J* = 1.6 Hz, 1H, 10-H), 8.24 (dd, *J* = 8.8 Hz, *J*’ = 1.6 Hz, 1H, 8-H), 8.08 (d, *J* = 8.8 Hz, 1H, 7-H), 7.55 [dm, *J* = 8.4 Hz, 2H, 4-chlorophenyl 2(6)-H], 7.48 (dm, *J* = 8.4 Hz, 2H, 4-chlorophenyl 3(5)-H], 4.48 (q, *J* = 7.2 Hz, 2H, 9-CO_2_C*H*_2_CH_3_), 3.32 (m, 2H, 2-H_2_), 2.80 (t, *J* = 4.4 Hz, 2H, 4-H_2_), 2.00 (m, 2H, 3-H_2_), 1.43 (t, *J* = 7.2 Hz, 3H, 9-CO_2_CH_2_C*H*_3_), 1.42 [s, 9H, OC(C*H*_3_)_3_].

#### 2.1.12. 5-(4-Chlorophenyl)-3,4-dihydrobenzo[*h*][1,6]naphthyridine-9-carboxylic acid (**20**)

This compound was synthesized using the same conditions described for compound **9**. From a solution of ester **19** (800 mg, 1.71 mmol) in MeOH (4 mL) and 40% aq. KOH (3 mL), a residue was obtained and taken up in MeOH (8 mL) and then treated with an HCl solution in dioxane (4M solution, 2.5 mL) to finally afford the carboxylic acid **20** in the form of the hydrochloride salt (829 mg), which was used in the following step without further purification [36]: ^1^H NMR (400 MHz, DMSO-d_6_) δ (ppm): 8.57 (d, *J* = 1.6 Hz, 1H, 10-H), 8.17 (dd, *J* = 8.8 Hz, *J*’ = 1.6 Hz, 1H, 8-H), 7.57–7.46 [m, 5H, 7-H, 4-chlorophenyl 2(6)-H and 3(5)-H], 3.40 (m, 2H, 2-H_2_), 2.63 (t, *J* = 6.0 Hz, 2H, 4-H_2_), 1.73 (tt, *J* = *J’* = 6.0 Hz, 2H, 3-H_2_).

#### 2.1.13. *N*^1^-(Pyrimidin-2-yl)octane-1,8-diamine (**24**)

A mixture of 2-chloropyrimidine (500 mg, 4.37 mmol) and 1,8-octanediamine (1.10 g, 7.63 mmol) was stirred in a closed vessel at 137 °C overnight. The solvent was removed in vacuo and the crude was purified by silica gel column chromatography, eluting with DCM/Ammonia, ca. 7N solution in MeOH 90:10, to afford amine **24** (486 mg, 50% yield) as a brown oil [38]; *R*_f_ = 0.62 (DCM/ammonia, ca. 7N solution in MeOH 9:1); ^1^H NMR (400 MHz, CD_3_OD) δ (ppm): 8.26 [d, *J* = 4.8 Hz, 2H, pyrimidine 4(6)-H], 6.58 (t, *J* = 4.8 Hz, 1H, pyrimidine 5-H), 3.35 (m, 2H, 1-H_2_), 2.63 (t, *J* = 7.2 Hz, 2H, 8-H_2_), 1.62 (tt, *J* = *J’* = 7.2 Hz, 2-H_2_), 1.48 (tt, *J* = *J’* = 7.2 Hz, 7-H_2_), 1.42–1.34 (m, 8H, 3-H_2_, 4-H_2_, 5-H_2_, 6-H_2_).

#### 2.1.14. *N*^1^-[4-(2-Fluorophenyl)pyrimidin-2-yl]octane-1,8-diamine (**25**)

This compound was synthesized using the same conditions described for compound **24**, starting from a mixture of 2-chloro-4-(2-fluorophenyl)pyrimidine (500 mg, 2.40 mmol) and 1,8-octanediamine (1.10 g, 7.63 mmol). The resulting crude was purified by silica gel column chromatography, eluting with DCM/Ammonia, ca. 7N solution in MeOH 90:10, to afford amine **25** (370 mg, 49% yield) as a yellow oil; *R*_f_ = 0.48 (DCM/ammonia, ca. 7N solution in MeOH 9:1); IR (ATR-FTIR) ν_max_ (cm^−1^): 3251, 2936, 2860, 1605, 1545, 1456, 1076, 943; ^1^H NMR (400 MHz, CD_3_OD) δ (ppm): 8.30 (d, *J* = 5.2 Hz, 1H, 6′-H), 8.08 (dd, *J* = *J’* = 7.2 Hz, 1H, 5”-H), 7.51 (dddd, *J* = 8.4 Hz, *J’* = 7.2 Hz, *J”* = 5.2 Hz, *J”’* = 2.0 Hz, 1H, 4”-H), 7.31 (ddd, *J* = 7.6 Hz, *J’* = 7.2 Hz, *J”* = 1.2 Hz, 1H, 6”-H), 7.23 (ddd, *J* = 12.0 Hz, *J’* = 8.4 Hz, *J”* = 1.2 Hz, 1H, 3”-H), 7.05 (dd, *J* = 5.2 Hz, *J’* = 2.4 Hz, 1H, 5′-H), 3.45 (t, *J* = 7.2 Hz, 2H, 1-H_2_), 2.65 (t, *J* = 7.2 Hz, 2H, 8-H_2_), 1.67 (tt, *J* = *J’* = 7.2 Hz, 2H, 2-H_2_), 1.52–1.35 (m, 10H, 3-H_2_, 4-H_2_, 5-H_2_, 6-H_2_, 7-H_2_); ^13^C NMR (101 MHz, CD_3_OD) δ (ppm): 163.9 (C, C2′), 162.6 (C, d, *J* = 255 Hz, C2”), 159.3 (C + CH, C4′, C6′), 133.2 (CH, d, *J* = 9.1 Hz, C4”), 131.7 (CH, d, *J* = 2.0 Hz, C5”), 126.9 (C, d, *J* = 11.1 Hz, C1”), 125.6 (CH, d, *J* = 12.0 Hz, C6”), 117.3 (CH, d, *J* = 23.2 Hz, C3”), 110.9 (CH, C5′), 42.4 (CH_2_, C1), 42.2 (CH_2_, C8), 33.3 (CH_2_, C7), 30.6 (CH_2_), 30.5 (CH_2_), 30.4 (CH_2_) (C2, C4, C5), 28.0 (CH_2_), 27.9 (CH_2_) (C3, C6); HRMS (ESI-TOF) *m*/*z* calculated for [C_18_H_25_FN_4_ + H]^+^: 317. 2141, found 317.2136.

#### 2.1.15. *N*^1^-(7-Chloroquinolin-4-yl)octane-1,8-diamine (**26**)

This compound was synthesized using the same conditions described for compound **24**, starting from a mixture of 4,7-dichloroquinoline (800 mg, 4.04 mmol) and 1,8-octanediamine (1.10 g, 7.63 mmol). The resulting crude was purified by silica gel column chromatography, eluting with DCM/Ammonia, ca. 7N solution in MeOH 90:10, to afford amine **26** (799 mg, 65% yield) as a yellowish solid [39]; ^1^H NMR (400 MHz, CDCl_3_) δ (ppm): 8.52 (d, *J* = 5.2 Hz, 1H, 2′-H), 7.95 (d, *J* = 2.0 Hz, 1H, 8′-H), 7.65 (d, *J* = 9.2 Hz, 1H, 5′-H), 7.35 (d, *J* = 9.2 Hz, *J’* = 2.0 Hz, 1H, 6′-H), 6.41 (d, *J* = 5.2 Hz, 1H, 3′-H), 4.98 (t, *J* = 5.6 Hz, 1H, NH), 3.30 (dt, *J* = *J’* = 5.6 Hz, 2H, 1-H_2_), 2.68 (t, *J* = 7.2 Hz, 2H, 8-H_2_), 1.75 (tt, *J* = *J’* = 7.2 Hz, 2H, 2-H_2_), 1.50–1.31 (m, 10H, 3-H_2_, 4-H_2_, 5-H_2_, 6-H_2_, 7-H_2_).

#### 2.1.16. 5-(4-Chlorophenyl)-*N*-[8-(pyrimidin-2-ylamino)octyl]-1,2,3,4-tetrahydrobenzo [*h*][1,6]naphthyridine-9-carboxamide (**27**)

This compound was synthesized using the same conditions described for hybrid **15**. From a solution of amine **24** (300 mg, 1.35 mmol) and carboxylic acid **20** (1.00 g of crude product, 2.95 mmol) in dry DMF (3 mL), DIPEA (0.5 mL, 2.87 mmol), and a solution of HATU (561 mg, 1.48 mmol) in DMF (4 mL), hybrid **27** (497 mg, 68% yield) was obtained, as a yellow solid, after silica gel column chromatography purification, eluting with DCM/ammonia, ca. 7N solution in MeOH 90:10; *R*_f_ = 0.48 (DCM/ammonia, ca. 7N solution in MeOH 9:1).

The analytical sample of **27**·2HCl was prepared as described before, upon treatment with 5M HCl in isopropanol (1 mL), washing of the solid with pentane (3 × 2 mL) and drying in vacuo; mp 196–197 °C; IR (ATR-FTIR) ν_max_ (cm^−1^): 3373, 3232, 2920, 2861, 2795, 1587, 1320, 1091, 844; ^1^H NMR (400 MHz, CDCl_3_) δ (ppm): 8.45 (br s, 1H, 10-H), 8.26 [d, *J* = 4.8 Hz, 2H, 4”(6”)-H], 7.89 (d, *J* = 8.8 Hz, 1H, 7-H), 7.85 (dd, *J* = 8.8 Hz, *J’* = 1.2 Hz, 1H, 8-H), 7.45 (dm, *J* = 8.4 Hz, 2H), 7.38 (dm, *J* = 8.4 Hz, 2H) [4-chlorophenyl 2(6)-H and 3(5)-H], 6.71 (t, *J* = 4.8 Hz, NH), 6.50 (t, *J* = 4.8 Hz, 1H, 5”-H), 6.13 (br s, 1H, NH), 5.23 (t, *J* = 4.8 Hz, 1H, NH), 3.51–3.43 (m, 4H, 2-H_2_, 8′-H_2_), 3.38 (dt, *J* = *J’* = 6.4 Hz, 2H, 1′-H_2_), 2.70 (t, *J* = 6.0 Hz, 2H, 4-H_2_), 1.88 (tt, *J* = *J’* = 6.0 Hz, 2H, 3-H_2_), 1.64–1.55 (m, 4H, 2′-H_2_, 7′-H_2_), 1.38–1.30 (m, 8H, 3′-H_2_, 4′-H_2_, 5′-H_2_, 6′-H_2_); ^13^C NMR (101 MHz, CDCl_3_) δ (ppm): 167.0 (C, 9-CONH), 162.6 (C, C2”), 159.3 (C, C10b), 158.2 [2CH, C4”(6”)], 148.2 (C, C5), 147.5 (C, C6a), 138.8 (C, 4-chlorophenyl C4), 134.5 (C, C9), 130.3 (C, chlorophenyl C1), 129.9 (2CH), 129.1 (2CH) [4-chlorophenyl C2(6) and C3(5)], 128.5 (CH, C8), 126.1 (CH, C10), 120.6 (CH, C7), 116.6 (C, C10a), 110.5 (CH, C5”), 108.2 (C, C4a), 50.9 (CH_2_, C8′), 41.6 (CH_2_, C2), 41.5 (CH_2_, C1′), 29.7 (CH_2_), 29.6 (CH_2_) (C2′, C7′), 29.28 (CH_2_), 29.27 (CH_2_) (C4′, C5′), 27.0 (CH_2_), 26.9 (CH_2_) (C3′, C6′), 25.5 (CH_2_, C4), 20.7 (CH_2_, C3); HRMS (ESI-TOF) *m*/*z* calculated for [C_31_H_36_^35^ClN_6_O + H]^+^: 543.2634, found 543.2639.

#### 2.1.17. 5-(4-Chlorophenyl)-*N*-{8-{[4-(2-fluorophenyl)pyrimidin-2-yl]amino}octyl}-1,2,3,4-tetrahydrobenzo[*h*][1,6]naphthyridine-9-carboxamide (**28**)

This compound was synthesized using the same conditions described for hybrid **15**. From a solution of amine **25** (71 mg, 0.22 mmol) and carboxylic acid **20** (230 mg of crude product, 2.95 mmol) in dry DMF (2 mL), DIPEA (0.2 mL, 1.15 mmol), and a solution of HATU (128 mg, 0.34 mmol) in DMF (4 mL), hybrid **28** (120 mg, 84% yield) was obtained, as a yellow solid, after silica gel column chromatography purification, eluting with DCM/ammonia, ca. 7N solution in MeOH 90:10; *R*_f_ = 0.44 (DCM/ammonia, ca. 7N solution in MeOH 9:1).

The analytical sample of **28**·2HCl was prepared as described before, upon treatment with 5M HCl in isopropanol (1 mL), washing of the solid with pentane (3 × 2 mL) and drying in vacuo; mp 198–199 °C; IR (ATR-FTIR) ν_max_ (cm^−1^): 3450, 3136, 2933, 2863, 1644, 1579, 1324, 1089, 824; ^1^H NMR (400 MHz, CDCl_3_) δ (ppm): 8.48 (br s, 1H, 10-H), 8.32 (br d, *J* = 4.8 Hz, 1H, 6”-H), 8.06 (dd, *J* = *J’* = 7.2 Hz, 1H, 5”’-H), 7.91 (br d, *J* = 8.8 Hz, 1H, 7-H), 7.85 (dd, *J* = 8.8 Hz, *J’* = 1.2 Hz, 1H, 8-H), 7.47 (dm, *J* = 8.8 Hz, 2H), 7.39 (dm, *J* = 8.8 Hz, 2H) [4-chlorophenyl 2(6)-H and 3(5)-H], superimposed 7.39 (m, 1H, 4”’-H), 7.23 (ddd, *J* = *J’* = 7.6 Hz, *J”* = 0.8 Hz, 1H, 6”’-H), 7.13 (ddd, *J* = 11.6 Hz, *J’* = 8.4 Hz, *J”* = 0.8 Hz, 1H, 3”’-H), 7.04 (dd, *J* = 4.8 Hz, *J’* = 2.0 Hz, 1H, 5”-H), 6.76 (t, *J* = 4.8 Hz, 1H, NH), 6.00 (br s, 1H, NH), 5.31 (t, *J* = 6.0 Hz, 1H, NH), 3.47–3.41 (m, 6H, 2-H_2_, 1′-H_2_, 8′-H_2_), 2.68 (t, *J* = 6.0 Hz, 2H, 4-H_2_), 2.30 (s, 1H, NH), 1.84 (tt, *J* = *J’* = 6.0 Hz, 2H, 3-H_2_), 1.59 (m, 4H, 2′-H_2_, 7′-H_2_), 1.41–1.31 (m, 8H, 3′-H_2_, 4′-H_2_, 5′-H_2_, 6′-H_2_); ^13^C NMR (101 MHz, CDCl_3_) δ (ppm): 167.1 (C, 9-CONH), 162.8 (C, C2”), 161.2 (C, d, *J* = 253 Hz, C2”’), 159.8 (C, C4”), 158.5 (CH, C6”), 148.0 (C, C10b), 147.8 (C, C5), 147.0 (C, 6a), 139.3 (C, 4-chlorophenyl C4), 134.3 (C, C9), 131.8 (CH, d, *J* = 9.1 Hz, C4”’), 130.6 (CH, d, *J* = 2.0 Hz, C5”’), 130.3 (2CH), 128.5 (2CH) [4-chlorophenyl C2(6) and C3(5)], 129.8 (C, 4-chlorophenyl C1), 129.7 (CH, C8), 125.8 (C, d, *J* = 10.1 Hz, C1”’), 124.6 (CH, C10), 120.6 (CH, C7), 116.7 (C, C10a), 116.5 (CH, d, *J* = 23.2 Hz, C3”’), 110.7 (C, C4a), 108.2 (CH, C5”), 41.6 (CH_2_, C8′), 40.4 (CH_2_, C2), 38.8 (CH_2_, C1′), 29.8 (CH_2_), 29.7 (CH_2_) (C2′, C7′), 29.3 (2CH_2_), (C4′, C5′), 27.03 (CH_2_), 26.97 (CH_2_) (C3′, C6′), 25.6 (CH_2_, C4), 20.8 (CH_2_, C3); HRMS (ESI-TOF) *m*/*z* calculated for [C_37_H_38_^35^ClFN_6_O + H]^+^: 637.3377, found 637.3398.

#### 2.1.18. 5-(4-Chlorophenyl)-*N*-{8-[(7-chloroquinolin-4-yl)amino]octyl}-1,2,3,4-tetrahydrobenzo[*h*][1,6]naphthyridine-9-carboxamide (**29**)

This compound was synthesized using the same conditions described for hybrid **15**. From a solution of amine **26** (200 mg, 0.65 mmol) and carboxylic acid **20** (1.00 g of crude product, 1.96 mmol) in dry DMF (3.5 mL), DIPEA (0.3 mL, 1.72 mmol), and a solution of HATU (373 mg, 0.98 mmol) in DMF (3 mL), hybrid **29** (165 mg, 40% yield) was obtained, as a yellow solid, after silica gel column chromatography purification, eluting with DCM/ammonia, ca. 7N solution in MeOH 90:10; *R*_f_ = 0.44 (DCM/ammonia, ca. 7N solution in MeOH 9:1).

The analytical sample of **29**·2HCl was prepared as described before, upon treatment with 5M HCl in isopropanol (1 mL), washing of the solid with pentane (3 × 2 mL) and drying in vacuo; mp 198–199 °C; IR (ATR-FTIR) ν_max_ (cm^−1^): 3450, 3136, 2933, 2863, 1644, 1579, 1324, 1089, 824; ^1^H NMR (400 MHz, CD_3_OD) δ (ppm): 9.02 (d, *J* = 1.2 Hz, 1H, 10-H), 8.46 (d, *J* = 9.2 Hz, 1H, 5”-H), 8.38 (d, *J* = 7.2 Hz, 1H, 8-H), 8.27 (dd, *J* = 9.2 Hz, *J’* = 2.0 Hz, 1H, 6”-H), 7.90–7.87 (m, 2H, 7-H, 8”-H), 7.69–7.66 [m, 5H, 2”-H, 4-chlorophenyl 2(6)-H and 3(5)-H], 6.88 (d, *J* = 7.2 Hz, 1H, 3”-H), 3.71 (t, *J* = 5.6 Hz, 2H, 2-H_2_), 3.60 (t, *J* = 7.4 Hz, 2H, 8′-H_2_), 3.45 (t, *J* = 7.2 Hz, 2H, 1′-H_2_), 2.75 (t, *J* = 6.2 Hz, 2H, 4-H_2_), 1.98 (tt, *J* = *J’* = 5.6 Hz, 2H, 3-H_2_), 1.82 (tt, *J* = *J’* = 7.2 Hz, 2H, 7′-H_2_), 1.71 (tt, *J* = *J’* = 6.8 Hz, 2H, 2′-H_2_), 1.52–1.44 (m, 8H, 3-‘H_2_, 4′-H_2_, 5′-H_2_, 6′-H_2_); ^13^C NMR (101 MHz, CD_3_OD) δ (ppm): 168.0 (C, 9-CONH), 157.5 (C, C4”), 155.7 (C, C10b), 151.5 (CH, C2”), 143.7 (C, C5), 140.9 (C, C8a”), 140.2 (C, C6a), 140.0 (C, C7”), 138.2 (C, 4-chlorophenyl C-4), 133.5 (C, C-9), 132.7 (CH, C8), 132.3 (C, 4-chlorophenyl C1), 131.9 (2CH), 130.4 (2CH) [4-chlorophenyl C2(6) and C3(5)], 128.6 (CH, C8”), 126.1 (CH, C5”), 123.6 (CH, C10), 121.0 (CH, C7), 120.3 (CH, C6”), 116.9 (C, C4a”), 116.2 (C, C10a), 109.8 (C, C4a), 99.7 (CH, C3”), 44.9 (CH_2_, C8′), 43.8 (CH_2_, C2), 41.3 (CH_2_, C1′), 30.4 (CH_2_), 30.2 (2CH_2_), 29.1 (CH_2_) (C2′, C4′, C5′, C7′), 28.0 (CH_2_), 27.9 (CH_2_) (C3′, C6′), 25.0 (CH_2_, C4), 20.0 (CH_2_, C3); HRMS (ESI-TOF) *m*/*z* calculated for [C_36_H_37_^35^Cl_2_N_5_O + H]^+^: 626.2972, found 626.2988.

#### 2.1.19. *N*^1^-(6-Chloro-1,2,3,4-tetrahydroacridin-9-yl)propane-1,3-diamine (**31**)

This compound was synthesized using the same conditions described for compound **14**, starting from a mixture of chloroquinoline **12** (500 mg, 1.98 mmol), 1,3-propanediamine (0.84 mL, 9.97 mmol), KI (33 mg, 0.20 mmol), and phenol (373 mg). The resulting crude was purified with pre-packed silica gel cartridges-RediSep^®^ onto Isco-CombiFlash^®^ Companion^®^ (DCM/ammonia, ca. 7N solution in methanol 90:10), to afford amine **31** (278 mg, 48% yield) as a brown oil [40]; *R*_f_ = 0.51 (DCM/ammonia, ca. 7N solution in MeOH 9:1); ^1^H NMR (400 MHz, CDCl_3_) δ (ppm): 7.94 (d, *J* = 9.2 Hz, 1H, 8′-H), 7.87 (d, *J* = 2.0 Hz, 1H, 5′-H), 7.26 (dd, *J* = 9.2 Hz, *J*’ = 2.0 Hz, 1H, 7′-H), 3.62 (t, *J* = 6.8 Hz, 2H, 1-H_2_), 3.02 (t, *J* = 6.0 Hz, 2H, 4′-H_2_), 2.92 (t, *J* = 6.8 Hz, 2H, 3-H_2_), 2.68 (t, *J* = 6.0 Hz, 2H, 1′-H_2_), 1.92–1.89 (m, 4H, 2′-H_2_, 3′-H_2_), 1.80 (tt, *J* = *J’* = 6.8 Hz, 2H, 2-H_2_).

#### 2.1.20. 4-Amino-*N*-{3-[(6-chloro-1,2,3,4-tetrahydroacridin-9-yl)amino]propyl}butanamide (**33**)

The amine **31** (570 mg, 1.97 mmol) was coupled to *N*-Boc-protected 4-aminobutyric acid, **32** (400 mg, 1.97 mmol), using the same conditions described for hybrid **15**, with DIPEA (0.86 mL, 4.92 mmol) and a solution of HATU (1.12 g, 2.95 mmol) in DMF (1 mL). The corresponding *N*-Boc-protected amide (1.08 g, quantitative yield) was obtained as an orange solid, after silica gel column chromatography purification, eluting with DCM/ammonia, ca. 7N solution in MeOH 90:10.

The *N*-Boc-protected amide (510 mg, 1.07 mmol) was treated with 4N HCl in dioxane (3 mL) and the reaction mixture was stirred at room temperature overnight. The solvent was evaporated to dryness and then the residue was taken up in MeOH (6 mL), diluted with 2N NaOH (3 mL), until pH 8–9, and was extracted with DCM (3 × 20 mL). The combined organic layers were dried over anhydrous Na_2_SO_4_, filtered and concentrated under reduced pressure to provide amine **33** (229 mg, 57% yield) as a yellow oil; IR (ATR-FTIR) ν_max_ (cm^−1^): 3396, 3263, 2942, 1632, 1574, 1516, 1453, 1249, 1092, 831; ^1^H NMR (400 MHz, DMSO-d_6_) δ (ppm): 8.43 (d, *J* = 9.2 Hz, 1H, 8”-H), 8.21 (t, *J* = 5.6 Hz, 1H, 1′-NH), superimposed in part 8.02 (m, 1H, 3′-NH), 8.00 (d, *J* = 2.0 Hz, 1H, 5”-H), 7.95 (br s, 2H, 4-NH_2_), 7.59 (dd, *J* = 9.2 Hz, *J’* = 2.0 Hz, 1H, 7”-H), 3.86 (dt, *J* = *J’* = 6.8 Hz, 2H, 3′-H_2_), 3.13 (m, 2H, 1′-H_2_), 2.99 (m, 2H, 4”-H_2_), 2.75 (m, 2H, 4-H_2_), 2.66 (m, 2H, 1”-H_2_), 2.17 (t, *J* = 7.2 Hz, 2H, 2-H_2_), superimposed in part 1.85 (tt, *J* = *J’* = 6.8 Hz, 2H, 2′-H_2_), 1.83 (m, 4H, 2”-H_2_, 3”-H_2_), 1.74 (tt, *J* = *J’* = 7.2 Hz, 2H, 3-H_2_); ^13^C NMR (101 MHz, DMSO-d_6_) δ (ppm): 171.6 (C, C1), 155.6 (C, C9”), 151.1 (C, C4a”), 138.7 (C, C10a”), 137.1 (C, C6”), 127.6 (CH, C8”), 125.3 (CH, C7”), 118.0 (CH, C5”), 114.1 (C, C8a”), 111.8 (C, C9a”), 44.7 (CH_2_, C3′), 38.5 (CH_2_, C4), 35.6 (CH_2_, C1′), 32.1 (CH_2_, C2), 29.8 (CH_2_, C2′), 27.9 (CH_2_, C4”), 23.9 (CH_2_, C1”), 23.2 (CH_2_, C3), 21.3 (CH_2_, C2”), 20.2 (CH_2_, C3”); HRMS (ESI-TOF) *m*/*z* calculated for [C_20_H_27_^35^ClN_4_O + H]^+^: 375.1946, found 375.1957.

#### 2.1.21. *N*-4-{{3-[(6-Chloro-1,2,3,4-tetrahydroacridin-9-yl)amino]propyl}amino}-4-oxobutyl}-5-(4-chlorophenyl)-1,2,3,4-tetrahydrobenzo[*h*][1,6]naphthyridine-9-carboxamide (**34**)

This compound was synthesized using the same conditions described for hybrid **15**. From a solution of amine **33** (500 mg, 1.33 mmol) and carboxylic acid **20** (700 mg of crude product, 1.60 mmol) in dry DMF (3.5 mL), DIPEA (0.2 mL, 1.15 mmol), and a solution of HATU (290 mg, 0.76 mmol) in DMF (2 mL), hybrid **34** (54 mg, 6% yield) was obtained, as a light brown solid, after silica gel column chromatography purification, eluting with DCM/ammonia, ca. 7N solution in MeOH 90:10; *R*_f_ = 0.77 (EtOAc/MeOH 9:1).

The analytical sample of **34**·2HCl was prepared as described before, upon treatment with 5M HCl in isopropanol (1 mL), washing of the solid with pentane (3 × 2 mL) and drying in vacuo; mp 205–206 °C; IR (ATR-FTIR) ν_max_ (cm^−1^): 3473, 3013, 2919, 2872, 1631, 1535, 1323, 1089, 800; ^1^H NMR (400 MHz, CD_3_OD) δ (ppm): 8.50 (d, *J* = 2.0 Hz, 1H, 10-H), 8.04 (d, *J* = 9.2 Hz, 1H, 8”’-H), 7.94 (dd, *J* = 8.8 Hz, *J’* = 2.0 Hz, 1H, 8-H), 7.77 (d, *J* = 8.8 Hz, 1H, 7-H), 7.68 (d, *J* = 2.0 Hz, 1H, 5”’-H), 7.47 [m, 4H, 4-chlorophenyl 2(6)-H and 3(5)-H], 7.26 (dd, *J* = 9.2 Hz, *J’* = 2.0 Hz, 1H, 7”’-H), 3.54 (t, *J* = 6.8 Hz, 2H, 3”-H_2_), 3.50–3.44 (m, 4H, 1′-H_2_, 1”-H_2_), 3.27 (t, *J* = 6.4 Hz, 2H, 2-H_2_), 2.91 (t, *J* = 5.4 Hz, 2H, 4”’-H_2_), 2.67 (t, *J* = 5.2 Hz, 2H, 1”’-H_2_), 2.62 (t, *J* = 6.4 Hz, 2H, 4-H_2_), 2.34 (t, *J* = 7.2 Hz, 2H, 3′-H_2_), 1.96 (t, *J* = 6.4 Hz, 2H, 3-H_2_), 1.88–1.77 (m, 8H, 2′-H_2_, 2”-H_2_, 2”’-H_2_, 3”’-H_2_); ^13^C NMR (101 MHz, CD_3_OD) δ (ppm): 176.1 (C, C4′), 169.7 (C, 9-CONH), 160.3 (C, C9”’), 159.5 (C, C10b), 153.5 (C, C4a”’), 150.5 (C, C5), 148.5 (C, C10a”’), 147.5 (C, C6a), 140.1 (C, C6”’), 135.9 (C, 4-chlorophenyl C4), 135.5 (C, C9), 131.1 (C, C5a), 131.1 (2CH), 129.4 (2CH) [4-chlorophenyl C2(6) and C3(5)], 128.5 (CH, C8”’), 128.1 (CH, C8), 126.5 (CH, C7”’), 125.8 (C, C9a”’), 125.3 (CH, C10), 122.4 (CH, C7), 119.0 (CH, C5”’), 117.7 (C, C10a), 116.6 (C, C8a”), 108.8 (C, C4a), 46.4 (CH_2_, C3”), 42.3 (CH_2_, C2), 40.8 (CH_2_, C1′), 37.5 (CH_2_, C1”), 34.7 (CH_2_, C3′), 33.7 (CH_2_, C2′), 31.9 (CH_2_, C2”), 26.6 (CH_2_, C4”’), 26.4 (CH_2_, C4), 26.0 (CH_2_, C1”’), 23.8 (CH_2_, C2”’), 23.3 (CH_2_, C3”’), 21.4 (CH_2_, C3); HRMS (ESI-TOF) *m*/*z* calculated for [C_39_H_41_^35^Cl_2_N_6_O_2_ + H]^+^ 695.2663, found 695.2654.

#### 2.1.22. *N*-(6-Chloro-1,2,3,4-tetrahydroacridin-9-yl)-3,6-dioxaoctane-1,8-diamine (**36**)

This compound was synthesized using the same conditions described for compound **24**, starting from a mixture of chloroquinoline **12** (1.00 g, 3.97 mmol) and ethylene glycol bis(2-aminoethyl)ether (2.20 g, 14.8 mmol). The resulting crude was purified using a pre-packed silica gel cartridge-RediSep^®^ onto Isco-CombiFlash^®^ Companion^®^ (DCM/ammonia, ca. 7N solution in methanol 9:1), to afford amine **36** (65 mg, 5% yield) as a brown oil [41]; ^1^H NMR (400 MHz, CDCl_3_) δ (ppm): 7.91 (d, *J* = 7.2 Hz, 1H, 8′-H), 7.89 (d, *J* = 1.6 Hz, 1H, 5′-H), 7.29 (dd, *J* = 7.2 Hz, *J’* = 1.6 Hz, 1H, 7′-H), 3.67 (m, 4H), 3.62 (m, 2H), 3.59 (m, 2H), 3.53 (t, *J* = 4.2 Hz, 2H) (1-H_2_, 2-H_2_, 4-H_2_, 5-H_2_, 7-H_2_), 3.04 (t, *J* = 4.8 Hz, 2H, 4′-H_2_), 2.89 (t, *J* = 4.2 Hz, 2H, 8-H_2_), 2.74 (t, *J* = 4.8 Hz, 2H, 1′-H_2_), 1.93–1.89 (m, 4H, 2′-H_2_, 3′-H_2_).

#### 2.1.23. *N*-{8-[(6-Chloro-1,2,3,4-tetrahydroacridin-9-yl)amino]-3,6-dioxaoctyl}-5-(4-chlorophenyl)-1,2,3,4-tetrahydrobenzo[*h*][1,6]naphthyridine-9-carboxamide (**37**)

This compound was synthesized using the same conditions described for hybrid **15**. From a solution of amine **36** (500 mg, 1.37 mmol) and carboxylic acid **20** (1.40 g of crude product, 4.12 mmol) in dry DMF (3.5 mL), DIPEA (0.6 mL, 3.44 mmol), and a solution of HATU (784 mg, 2.06 mmol) in DMF (3 mL), hybrid **37** (145 mg, 15% yield) was obtained, as a light brown solid, after silica gel column chromatography purification, eluting with DCM/ammonia, ca. 7N solution in MeOH 90:10; *R*_f_ = 0.63 (DCM/ammonia, ca. 7N solution in MeOH 95:5).

The analytical sample of **37**·2HCl was prepared as described before, upon treatment with 5M HCl in isopropanol (1 mL), washing of the solid with pentane (3 × 2 mL) and drying in vacuo; mp 210–211 °C; IR (ATR-FTIR) ν_max_ (cm^−1^): 3274, 3232, 2823, 2861, 2795, 1651, 1573, 1444, 1092, 844; ^1^H NMR (400 MHz, CD_3_OD) δ (ppm): 8.41 (d, *J* = 2.0 Hz, 1H, 10-H), 7.87 (dd, *J* = 8.8 Hz, *J’* = 2.0 Hz, 1H, 8-H), 7.86 (d, *J* = 9.2 Hz, 1H, 8”-H), 7.62 (d, *J* = 8.8 Hz, 1H, 7-H), 7.58 (d, *J* = 2.0 Hz, 1H, 5”-H), 7.48–7.42 [m, 4H, 4-chlorophenyl 2(6)-H and 3(5)-H], 7.15 (dd, *J* = 9.2 Hz, *J’* = 2.0 Hz, 1H, 7”-H), 3.73 (t, *J* = 5.2 Hz, 2H, 8′-H_2_), 3.69–3.61 (m, 8H, 2′-H_2_, 4′-H_2_, 5′-H_2_, 7′-H_2_), 3.57 (dt, *J* = *J’* = 4.8 Hz, 2H, 1′-H_2_), 3.40 (t, *J* = 5.4 Hz, 1H, 2-H_2_), 2.82 (t, *J* = 5.4 Hz, 2H, 4”-H_2_), 2.56–2.52 (m, 4H, 4-H_2_, 1”-H_2_), 1.97–1.73 (m, 6H, 3-H_2_, 2”-H_2_, 3”-H_2_); ^13^C NMR (101 MHz, CD_3_OD) δ (ppm): 167.6 (C, 9-CONH), 157.8 (C, C9”), 155.6 (C, C10b), 152.2 (C, C4a”), 151.5 (C, C5), 140.3 (C, C10a”), 140.1 (C, C6a), 140.0 (C, C6”), 138.4 (C, 4-chlorophenyl C4), 132.9 (C, C9), 132.3 (CH, C8), 132.2 (C, 4-chlorophenyl C1), 131.9 (2CH), 130.4 (2CH) [4-chlorophenyl C2(6) and C3(5)], 128.9 (CH, C8”), 126.7 (C, C7”), 123.7 (CH, C10), 120.9 (CH, C7), 118.9 (CH, C5”), 116.0 (C, C10a), 115.4 (C, C8a”), 113.8 (C, C9a”), 109.8 (C, C4a), 71.3 (CH_2_), 71.1 (CH_2_), 70.3 (CH_2_), 70.0 (CH_2_) (C2′, C4′, C5′, C7′), 42.9 (CH_2_, C2), 41.1 (2CH_2_, C1′, C8′), 29.3 (CH_2_, C4”), 25.1 (CH_2_, C4), 24.2 (CH_2_, C1”), 22.7 (CH_2_, C2”), 21.7 (CH_2_, C3”), 20.0 (CH_2_, C3); HRMS (ESI-TOF) *m*/*z* calculated for [C_38_H_40_^35^Cl_2_N_5_O_3_ + H]^+^: 684.2503, found 684.2523.

### 2.2. Biological Profiling of the Target Compounds

#### 2.2.1. In Vitro Evaluation of hAChE and hBChE Inhibitory Activity

The inhibitory activity of the new hybrid compounds against human cholinesterases, namely human recombinant AChE and BChE from human serum (Merck Millipore, Milan, Italy), was assessed by using Ellman’s spectrophotometric method [42]. Lyophilized hAChE and hBChE were reconstituted in 0.1 M potassium phosphate at pH 8.0, containing 0.1% Triton X-100 and 0.1% aqueous gelatin, respectively. Stock solutions of the test compounds (1 mM) were prepared in methanol and further diluted in the same solvent to obtain the desired working concentrations. The assay mixture consisted of 340 μM 5,5′-dithiobis(2-nitrobenzoic acid) (DTNB), 0.02 unit/mL of hAChE or hBChE, and 0.1 M potassium phosphate at pH 8.0, in the absence or presence of the test compounds. Assay solutions were preincubated at 37 °C for 20 min before the addition of the appropriate substrate, i.e., acetylthiocholine iodide or butyrylthiocholine iodide (final concentration 550 μM). Blank samples lacking enzyme were prepared to account for the non-enzymatic substrate hydrolysis. The increase in absorbance at 412 nm, corresponding to the formation of 5-thio-2-nitrobenzoate, was monitored using a Jasco V-530 double-beam spectrophotometer equipped with a thermostated cuvette holder, which was set at 37 °C. IC_50_ values were calculated from concentration–response curves generated using at least five inhibitor concentrations producing 20–80% inhibition. Results are expressed as mean ± SEM of at least two independent experiments, each performed in triplicate, and were analyzed using GraphPad Prism version 8.

#### 2.2.2. Aβ42 and Tau Aggregation Inhibition in *E. coli* Cells

For the overexpression of Aβ42, 10 mL of M9 minimal medium supplemented with kanamycin (50 μg/mL) was used to inoculate a colony of *E. coli* BL21 (DE3) competent cells carrying the pET28a plasmid (Novagen, Inc., Madison, WI, USA), which encodes the DNA sequence for Aβ42. After overnight incubation, the necessary volume to achieve a 1:500 dilution was transferred to fresh M9 minimal medium containing kanamycin (50 μg/mL) and thioflavin-S (Th-S, 250 µM) [43].

For the overexpression of tau, 10 mL of M9 minimal medium supplemented with glucose (0.5%), ampicillin (50 μg/mL), and chloramphenicol (12.5 μg/mL) was used to culture *E. coli* BL21 (DE3) cells carrying the pTARA plasmid, which contains the T7 phage RNA polymerase gene (T7RP) under the control of the PBAD promoter. These cells were subsequently transformed with the pRKT42 vector. In this case, after overnight incubation, the required volume for a 1:500 dilution was transferred to fresh M9 minimal medium containing glucose (0.5%), chloramphenicol (12.5 μg/mL), ampicillin (50 μg/mL), and Th-S (250 µM) [43].

The cultures were incubated overnight at 37 °C and 250 rpm until reaching an OD600 of 0.6. Then, 980 µL of the cultures were transferred to 1.5 mL Eppendorf tubes containing 10 µL of a DMSO solution with the new hybrids and 10 µL of either isopropyl 1-thio-β-D-galactopyranoside (IPTG, 100 mM) for Aβ42 overexpression or arabinose (25%) for tau overexpression, resulting in a final compound concentration of 10 µM. The cultures were further incubated overnight at 37 °C and 1400 rpm using a Thermomixer (Eppendorf, Hamburg, Germany). As a negative control (maximum Aβ42 or tau expression), the same DMSO volume without the test compounds was added. Additionally, non-induced samples (lacking IPTG or arabinose) were prepared as positive controls to assess the potential intrinsic toxicity of the compounds in the absence of Aβ42 or tau.

To evaluate the inhibitory effect of the new compounds on Aβ42 and tau aggregation in *E. coli* cells, a fluorescence assay was conducted using Th-S (2500 mM stock solution, T1892, Sigma, St. Louis, MO, USA) dissolved in double-distilled water (Milli-Q system, Millipore, Burlington, MA, USA) [43]. Measurements were performed with an Aminco Bowman Series 2 luminescence spectrophotometer (Aminco-Bowman AB2, SLM Aminco, Rochester, NY, USA) at 25 °C, within a wavelength range of 460–600 nm, with an excitation wavelength (λex) of 445 nm, emission wavelength (λem) of 485 nm, and slit widths of 4 nm. Data represent the average of 6 independent experiments, each conducted in duplicate. Steady-state fluorescence was tracked using CLARIOstar Plus (BMG LABTECH) with an excitation wavelength (λex) of 445 nm, emission wavelength (λem) of 485 nm, and absorbance using DTX 800 plate reader Multimode Detector equipped with Multimode Analysis Software (Beckman-Coulter, Brea, CA, USA) with a 535 ± 25 nm filter. The absorbance of the samples was assessed to confirm the correct bacterial growth, discarding the potential intrinsic toxicity of the dyes. DP-128, as a known Aβ42 and tau aggregation inhibitor, served as a positive control, demonstrating inhibition rates of 74.0 ± 4.1% and 68.0 ± 3.2% at 10 μM, respectively.

#### 2.2.3. Cytotoxicity

The human SH-SY5Y cell line (HD 104-057 and HD 104-004), generously provided by Dr. Coral Sanfeliu, was routinely maintained in DMEM media supplemented with 10% fetal bovine serum and 1× penicillin-streptomycin under a humidified 5% CO_2_ atmosphere at 37 °C.

Cell viability was evaluated using the CellTiter-Glo^®^ kit (Promega, Madison, WI, USA). Cells were seeded in 96-well plates at a density of 10,000 cells per well in 100 µL of media. For IC_50_ determination, 2-fold serial dilutions of the compounds, starting at 1 mM, were tested in duplicate across 11 concentrations. After 24 h, cell viability was measured following the manufacturer’s instructions. Luminescence was recorded using a CLARIOStar plate reader, Allmendgrün 8 (BMG LABTECH, Ortenberg, Germany) at room temperature, with an integration time of 1 s. The counts per second were normalized to the DMSO control. LD_50_ values were determined by fitting the data to a standard Hill slope inhibition equation using GraphPad Prism 10 software.

#### 2.2.4. Experimental Determination of In Vitro Brain Permeability

A parallel artificial membrane permeability assay for blood–brain barrier (PAMPA-BBB) [44] was used to determine in vitro the brain permeability of the newly synthesized compounds. The amount of the test compounds, dissolved in a mixture of PBS:EtOH 70:30, that passed across a lipid extract of porcine brain membrane (Avanti Research, Alabaster, AL, USA), used as an artificial BBB, was determined. The assay was validated by comparing the permeability (*Pe*) values of 14 reference drugs that were determined under the assay conditions employed in this work with those values reported in the literature (see Appendix A.1, Table A1). The lineal correlation between these sets of values led to the following equation: *Pe* (exp) = 1.593 *Pe* (lit) − 1.230 (R^2^ = 0.940), which, together with the limits for BBB permeation established by Di et al. [44], enabled the definition of the BBB permeability ranges. CNS + (high permeation) compounds have *Pe* (10^−6^ cm/s) values greater than 5.14; CNS − (low permeation) compounds have *Pe* (10^−6^ cm/s) values lower than 1.96; CNS ± (uncertain permeation) compounds have *Pe* (10^−6^ cm/s) values between 1.96 and 5.14. The experimental *Pe* values are given as mean ± SD of three different experiments, performed in triplicate.

#### 2.2.5. Experimental Determination of Aqueous Solubility

Ten millimolar stock solutions of the new hybrids were conveniently diluted in a 384-well transparent plate (Greiner 781801, Madrid, Spain) with a mixture of DMSO:PBS 1:99, to go from 300 to 0.1 μM concentration. The resulting solutions were incubated at 37 °C, and 2 h later, they were read in a NEPHELOstar Plus laser nephelometer (BMG LABTECH, Ortenberg, Germany). The solubility of the compounds was calculated by adjusting the results to a segmented regression. As reference compounds, progesterone and digoxin were used, with experimental solubility values under these assay conditions of 8.2 μM and 75.8 μM, respectively (described as 3.7 μM and 59.3 μM, [45] respectively).

#### 2.2.6. Experimental Determination of In Vitro Plasma Stability in Three Species

The stability of the novel hybrids in human (h), mouse (m), and rat (r) plasma (Seralab), from healthy donors, was determined in plates that contained 1 μM plasma solutions of the test compounds in a total volume of 100 μL. The plates were incubated at 37 °C for 0 and 60 min. Then, plasma proteins were precipitated upon the addition of 300 μL of acetonitrile. The plate was then centrifuged at 4000 g at 4 °C for 60 min. The supernatant was taken and the amount of remaining compound was analyzed by UPLC-MS/MS (UPLC QSM Waters Acquity, Milford, MA, USA): stationary phase, ACQUITY BEH C18 (1.7 µm, 50 mm × 2.1 mm, Milford, MA, USA); mobile phase, mixtures of component A (0.1% aqueous formic acid) and component B (0.1% formic acid solution in acetonitrile), with a flow 0.6 mL/min, using the following gradient: 100% A during 0.1 min, from 100% A to 100% B until t = 1 min, 100% B until t = 2 min, from 100% B to 100% A until t = 2.1 min, and then 100% A until t = 2.5 min. The percentage of the compound remaining unchanged was calculated from the MS peak areas.

#### 2.2.7. Experimental Determination of In Vitro Microsomal Stability in Three Species

The microsomal stability of the new hybrids in human (h), mouse (m), and rat (r) microsomes (TebuBio-Xenotech, Le Perray-en-Yvelines, France/Thermo-Fisher, Waltham, MA, USA) was determined in 96-well microplates containing the following components: 50 mM phosphate buffer Na/K pH 7.4 (301.2 μL (h)/298.4 μL (m)/307.5 μL (r)); 30 mM MgCl_2_, 10 mM NADP, 100 mM glucose-6-phosphate, 40 u/mL glucose-6-phosphate dehydrogenase as cofactors (163 μL for the 3 species); human microsomes (30.8 μL), mouse microsomes (33.6 μL), or rat microsomes (24.5 μL); and a solution of the new hybrids (5 μL), to get a final volume of 500 μL in all cases. The plates were incubated at 37 °C and 75 μL samples were taken at different times (0, 10, 20, 40, and 60 min). The samples were transferred to a microplate and were treated with 75 μL acetonitrile plus rolipram to inactivate the microsomes. Then, 30 μL of water containing 0.5% formic acid was added to improve the chromatographic conditions, keeping the samples at 4 °C. The plate with all the samples was centrifuged at 4600 g at 15 °C for 30 min. The supernatants were separated and the amount of remaining compound was analyzed by UPLC-MS/MS (UPLC QSM Waters Acquity): stationary phase, ACQUITY BEH C18 (1.7 µm, 50 mm × 2.1 mm, Waters); mobile phase, mixtures of component A (0.1% aqueous formic acid) and component B (0.1% formic acid solution in acetonitrile), with a flow 0.6 mL/min, using the following gradient: 95% A − 5% B during 0.1 min, from 95% A − 5% B to 100% B until t = 1 min, 100% B until t = 2.5 min, from 100% B to 95% A − 5% B until t = 2.6 min, and then 95% A − 5% B until t = 3 min. The metabolic stability of the novel hybrids was calculated from the logarithm of the remaining compound at each of the time points.

### 2.3. Molecular Modeling in AChE and BChE

The binding modes of DP-128 and compounds **15**, **16**, and **34** were explored by means of docking calculations conducted with rDock [46]. The crystallographic structures of AChE in complex with huprine W and fasciculin 2 (PDB id 4BDT [47]) and BChE in complex with a chlorotacrine-tryptophan multi-target inhibitor (PDB id 6I0C [48]) were used as starting structures to build the docking models. Building of the ligand models and standard protein preparation procedures, including the removal of solvent molecules, crystallization adjuvants and co-crystallized ligands, were performed with MOE2026 [49]. Standard protonation states at pH 7.4 were used for all sidechains in both proteins, except for Glu285, Glu450 and Glu452 in AChE that were modelled in their neutral form. In both cases, the docking cavity was defined using the reference ligand method, with a radius of 14 Å around an artificial molecule that fused the chemical structures of donepezil, *bis*(7)-tacrine and *syn*-TZ2PA6. Pharmacophoric restraints for a positive charge, an H-bond donor and a hydrophobic atom were placed at the CAS. Each ligand underwent 100 runs of the RDocks genetic algorithm and the poses were scored with the desolvation scoring function and ranked according to their total score.

## 3. Results and Discussion

### 3.1. Design of the DP-128 Analogues

**Figure 2:**
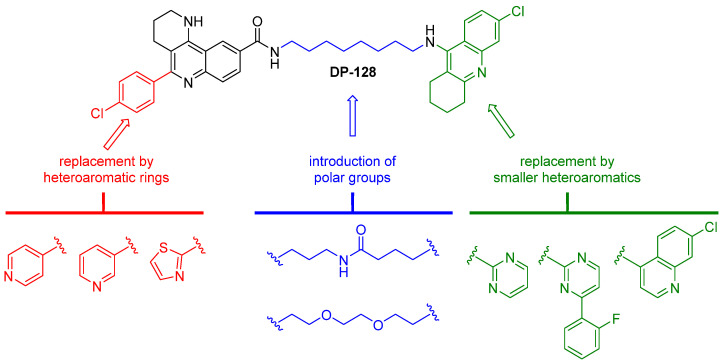
Design strategy of DP-128 analogues.

DP-128 was initially designed by molecular hybridization of two molecules with high affinity towards the catalytic and peripheral aromatic sites of AChE (CAS, PAS), i.e., 6-chlorotacrine [50] and 5-(4-chlorophenyl)-*N*-ethyl-1,2,3,4-tetrahydrobenzo[*h*][1,6]naphthyridine-9-carboxamide [36], respectively. Both ligands were connected through oligomethylene linkers of different lengths. Among that initial series of hybrids, the octamethylene-linked derivative (DP-128) turned out to be the best in terms of potent inhibitory activity of AChE, as the primary biological target, as well as Aβ42 and tau antiaggregating activity [27]. These results were in line with the findings in different structural families of multisite inhibitors of AChE, which featured two more or less extended π-systems connected by a tether of suitable length, for which we consistently found a significant Aβ42 and tau antiaggregating activity [51]. Thus, during the lead optimization we have either retained the octamethylene linker of DP-128 or have replaced it with functionalized linkers with the same length (see below). The new analogues have been designed by modifications in the structure of the two terminal ligands and within the linker, aimed at reducing lipophilicity and molecular weight, as previously mentioned. In an initial series, we have replaced the lipophilic 4-chlorophenyl group at position 5 of the 1,2,3,4-tetrahydrobenzo[*h*][1,6]naphthyridine moiety of DP-128 by more polar heteroaromatic rings. We chose 2-thiazolyl, 3-pyridyl, and 4-pyridyl groups (Figure 2), since pyridine and thiazole rings are frequently present in the structure of drugs, but other polar heterocycles like pyrimidine, pyridazine, pyrazine, oxazole, etc., would have also been good options. In another series, we have replaced the lipophilic tricyclic 6-chloro-1,2,3,4-tetrahydroacridine moiety of DP-128 by smaller, more polar mono- or bicyclic heteroaromatic systems (pyrimidin-2-yl, 7-chloroquinolin-4-yl, 4-(2-fluorophenyl)pyrimidin-2-yl). Given the high affinity of 6-chlorotacrine for AChE, it might be anticipated that its replacement by smaller heterocycles could result in a loss of AChE inhibitory potency. However, different families of potent homo- and heterodimeric AChE inhibitors have been described in which a tacrine unit was replaced by smaller 4-aminoquinoline or 4-aminopyridine moieties or that bear structurally related tetrahydroquinolinone systems [52,53]. Indeed, the 4-aminoquinoline core has been proposed as a suitable starting point for the design of multisite inhibitors of AChE [54]. Finally, we have introduced polar groups within the octamethylene linker of DP-128, namely 2 polyethylene glycol (PEG) groups or an amide.

In agreement with the design strategy, all the designed compounds are less lipophilic than the lead DP-128, with a decrease in the calculated log P values from −0.50 to −2.28 logarithmic units (calculated with Molinspiration [34]) or −0.99 to −2.84 logarithmic units (calculated with SwissADME [35]). They are also smaller, with a decrease in the molecular weight from −27 to −138 Da, with the sole exceptions of the analogues modified by the introduction of heteroatoms within the linker, in which the polarity was increased at the expense of a modest increase in molecular weight (by +4 and +15 Da) (see Appendix A.2, Table A2).

### 3.2. Synthesis of the DP-128 Analogues

**Scheme 1:**
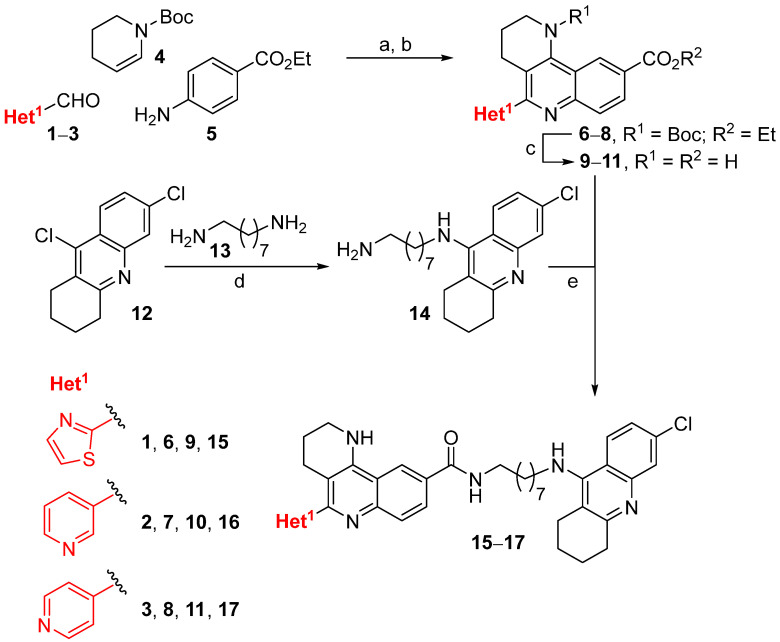
Synthesis of hybrids **15**–**17**. Reagents and conditions: (a) Yb(OTf)_3_, acetonitrile, room temperature, 3 days; (b) DDQ, dichloromethane, room temperature, overnight, 16% (**6**), 75% (**7**), 8% (**8**) overall yield over steps a and b, after silica gel column chromatography purification; (c) (1) 40% aq. KOH, MeOH, reflux, overnight, (2) 4M HCl in dioxane, room temperature, 30 min; (d) KI, phenol, 180 °C, 3 h, 19% (**14**), after silica gel column chromatography purification; (e) HATU, DIPEA, DMF, room temperature, overnight, 6% (**15**), 9% (**16**), 12% (**17**) overall yield over steps c and e.

The novel DP-128 analogues modified at position 5 of the 1,2,3,4-tetrahydrobenzo[*h*][1,6]naphthyridine moiety **15**–**17** were synthesized through a five-step convergent sequence that involved an initial multicomponent Povarov Yb(OTf)_3_-catalyzed reaction between the enamine **4**, the aniline **5** and the corresponding heteroaromatic aldehydes **1**–**3** (thiazole-2-carbaldehyde (**1**), pyridine-3-carbaldehyde (**2**), pyridine-4-carbaldehyde (**3**)), followed by DDQ oxidation of the resulting diastereomeric mixture of octahydrobenzo[*h*][1,6]naphthyridines to afford the *N*-Boc-protected 1,2,3,4-tetrahydrobenzo[*h*][1,6]naphthyridine esters **6**–**8** (Scheme 1). Alkaline hydrolysis of esters **6**–**8** with 40% aq. KOH in MeOH, followed by treatment of the reaction crude with 4M HCl in dioxane, yielded the carboxylic acids **9**–**11**, which were reacted with 8-aminooctyl-6-chlorotacrine **14** [37], which in turn was obtained by nucleophilic aromatic substitution reaction of the 6,9-dichloroacridine derivative **12** [55] with 1,8-octanediamine. The amide coupling of the carboxylic acids **9**–**11** with amine **14**, using HATU as the coupling reagent and DIPEA as the base, in dry DMF at room temperature afforded the final hybrids **15**–**17** in low yields after silica gel column chromatography purification (Scheme 1).

**Scheme 2:**
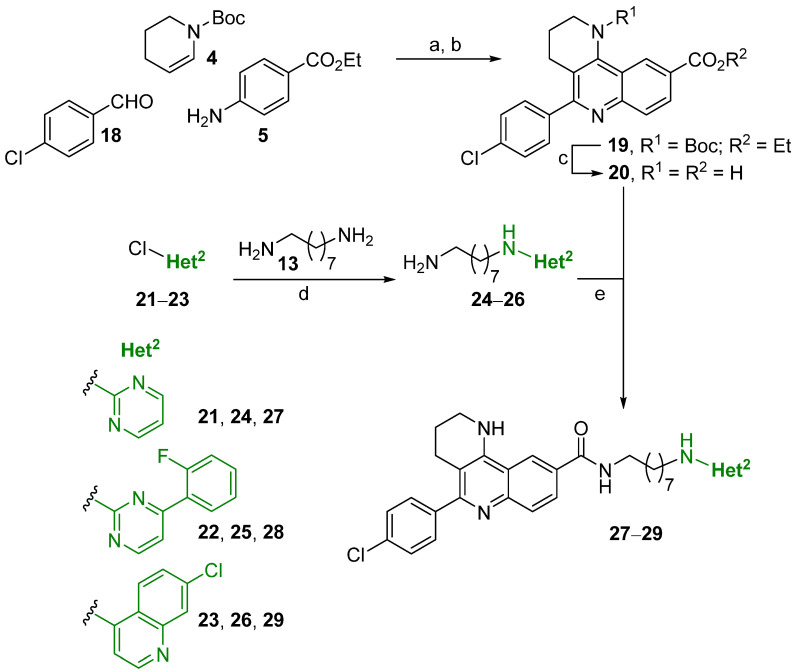
Synthesis of hybrids **27**–**29**. Reagents and conditions: (a) Yb(OTf)_3_, acetonitrile, room temperature, 3 days; (b) DDQ, dichloromethane, room temperature, overnight, 35% (**19**) overall yield over steps a and b, after silica gel column chromatography purification; (c) (1) 40% aq. KOH, MeOH, reflux, overnight, (2) 4M HCl in dioxane, room temperature, 30 min; (d) closed vessel, 137 °C, overnight, 50% (**24**), 49% (**25**), 65% (**26**), after silica gel column chromatography purification; (e) HATU, DIPEA, DMF, room temperature, overnight, 68% (**27**), 84% (**28**), 40% (**29**) overall yield over steps c and e.

For the synthesis of the DP-128 analogues modified at the 6-chloro-1,2,3,4-tetrahydroacridine moiety, a similar sequence was used. The Povarov reaction of enamine **4**, aniline **5**, and 4-chlorobenzaldehyde, **18**, followed by DDQ oxidation, ester hydrolysis, and *N*-Boc-deprotection, led to the carboxylic acid **20** [36]. Amination of 2-chloropyrimidine and 4-chloroquinoline reagents **21**–**23** with 1,8-octanediamine afforded the 8-aminooctylaminoheteroaromatics **24**–**26**, which were coupled with carboxylic acid **20** using HATU and DIPEA in dry DMF to afford the desired compounds **27**–**29** in moderate to high yields (Scheme 2).

**Scheme 3:**
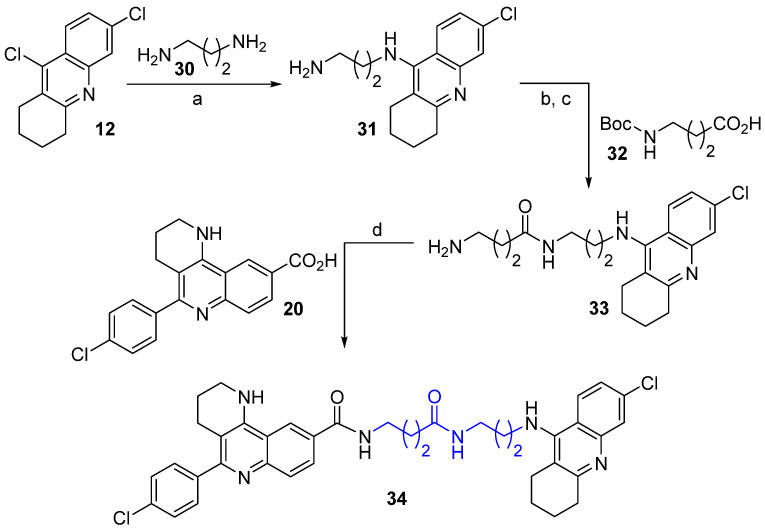
Synthesis of hybrid **34**. Reagents and conditions: (a) KI, phenol, 180 °C, 3 h, 48% (**31**), after silica gel column chromatography purification; (b) HATU, DIPEA, DMF, room temperature, overnight, 91% yield of *N*-Boc-protected intermediate; (c) 4M HCl in dioxane, room temperature, overnight, 57% yield (**33**); (d) HATU, DIPEA, DMF, room temperature, overnight, 6% yield (**34**).

The DP-128 analogues modified by the introduction of polar groups within the linker were prepared through 2–4-step synthetic sequences starting from 6,9-dichloroacridine **12**. Amination of **12** with 1,3-propanediamine, in the presence of a catalytic amount of KI in phenol at 180 °C for 3 h led to the 3-aminopropyl-6-chlorotacrine **31** [40], which was coupled with the commercially available *N*-Boc-protected 4-aminobutyric acid, **32**, using HATU and DIPEA in dry DMF, and then deprotected by treatment with 4M HCl in dioxane, to afford in moderate yield the amine **33**. Final amide coupling of the amine **33** with the carboxylic acid **20** led to the desired hybrid **34** (Scheme 3).

**Scheme 4:**
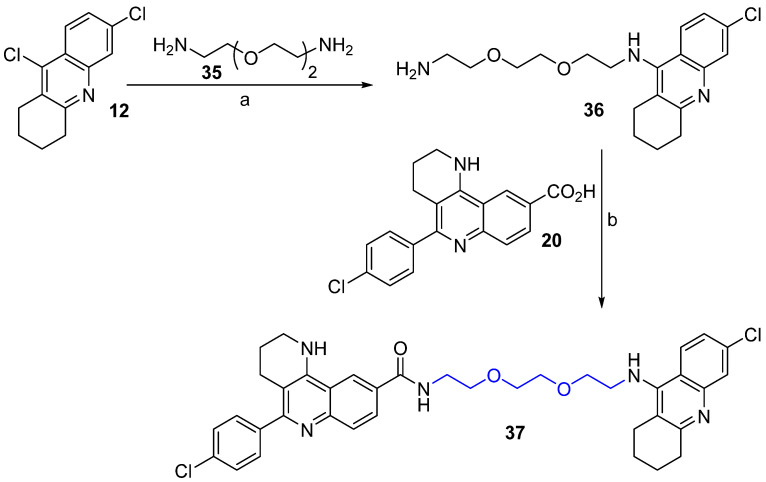
Synthesis of hybrid **37.** Reagents and conditions: (a) closed vessel, 137 °C, overnight, 6% yield (**36**), after silica gel column chromatography purification; (b) HATU, DIPEA, DMF, room temperature, overnight, 15% yield (**37**), after silica gel column chromatography purification.

Amination of **12** with ethylene glycol bis(2-aminoethyl)ether led to amine **36**, which was then subjected to amide coupling with carboxylic acid **20**, to afford the final hybrid **37** in 15% yield (Scheme 4).

The analytical samples of the novel DP-128 analogues subjected to biological evaluation were obtained by converting the hybrids **15**–**17**, **27**–**29**, **34**, and **37** into the corresponding hydrochloride salts through treatment with isopropanol or diethyl ether HCl solutions.

### 3.3. Evaluation of the Activity and Cytotoxicity of the DP-128 Analogues

#### 3.3.1. Anticholinesterase Activity: In Vitro Inhibition and Docking Studies

The primary pharmacological target of the lead compound DP-128 was AChE, as it was rationally designed as a dual binding site inhibitor capable of simultaneously engaging both the CAS and PAS. This binding mode is mediated by the 6-chlorotacrine moiety, which anchors within the CAS, and the benzonaphthyridine fragment, which extends toward and interacts with residues lining the PAS. Despite its design as an AChE inhibitor, DP-128 also displayed a potent inhibitory activity toward butyrylcholinesterase (BChE), another serine hydrolase responsible for the hydrolysis of the neurotransmitter acetylcholine. Since BChE activity increases in advanced AD stages, partially compensating for reduced AChE levels, BChE inhibition is also regarded as a therapeutically relevant strategy [56,57,58,59]. To assess whether the newly synthesized DP-128 analogues retained the anticholinesterase profile of the parent compound (IC_50_ hAChE = 2.06 nM; IC_50_ hBChE = 286 nM), their inhibitory activity was determined using Ellman’s spectrophotometric assay [42], using recombinant human AChE (hAChE) and BChE from human serum (hBChE). Consistent with the selectivity and potency profile of 6-chlorotacrine, all derivatives retaining the 6-chlorotacrine moiety showed strong hAChE inhibitory activity, with IC_50_ values in the single-digit nanomolar range (Table 1 and Supplementary Figure S1). In contrast, compounds **27**–**29,** in which the tetrahydroacridine core of 6-chlorotacrine was replaced by simplified heteroaromatic systems, showed only weak hAChE inhibitory activity. Among the active derivatives, most compounds showed higher potency than the reference 6-chlorotacrine (IC_50_ hAChE = 14.5 nM). Notably, compound **34** (IC_50_ hAChE = 1.31 nM) emerged as the most potent analogue, slightly outperforming the lead DP-128, whereas compounds **15**–**17** displayed IC_50_ values around 5 nM. Compound **37** (IC_50_ hAChE = 39.4 nM), although approximately one order of magnitude less potent than **34** and **15**–**17**, still qualifies as a strong nanomolar inhibitor.

Overall, these results indicate that the presence of the 6-chlorotacrine moiety is necessary for maintaining a high hAChE inhibitory activity, while structural modifications at the benzonaphthyridine unit exert only marginal effects on activity. In future endeavors, other heterocyclic systems like chroman and coumarin might be considered. Actually, they have already been used, as PAS-binding moieties, in a number of multisite AChE inhibitors in combination with a tacrine unit, not replacing it [41,60,61,62,63,64]. However, the high affinity of the tetrahydrobenzonaphthyridine fragment of DP-128 toward the PAS of AChE [36] might direct the binding of coumarin or chroman moieties toward the CAS, thereby making it reasonable to replace the lipophilic 6-chlorotacrine unit of DP-128 by these smaller heterocycles. Likewise, other smaller rings that are present in currently marketed AChE inhibitors, such as the *N*-benzylpiperidine ring of donepezil, might be used as the CAS-binding moiety in combination with the tetrahydrobenzonaphthyridine fragment of DP-128, as it has been used in a number of families of multisite AChE inhibitors [65,66,67].

As for the changes within the linker region of DP-128, the two studied structural modifications were generally well tolerated. In particular, the introduction of an amide proved to be favorable, leading to the highest potency (compound **34**). In contrast, replacement with a PEG-based linker slightly reduced potency to the two-digit nanomolar range. This result is consistent with previous reports on other families of 6-chlorotacrine-based hybrids, in which PEG spacers were associated with modest reductions in AChE inhibition compared to the corresponding oligomethylene-tethered congeners [41].

6-Chlorotacrine is well recognized for its marked selectivity toward AChE over BChE inhibition, due to the presence of the chlorine atom at position 6 of the tacrine scaffold, which enables favorable hydrophobic interactions within a hydrophobic pocket at the CAS of AChE [68]. Structural and crystallographic studies have shown that this substitution optimally complements the narrower and more aromatic AChE gorge. Conversely, the bulkier and more flexible active site of BChE, characterized by key residue substitutions, does not accommodate the 6-chloro substituent as efficiently, leading to suboptimal interactions and possible steric clashes [69]. This selectivity profile is commonly shared by the 6-chlorotacrine-based hybrid compounds, including DP-128, and it is likewise observed in the newly synthesized DP-128 analogues. Nevertheless, as observed for AChE inhibition, the presence of the 6-chlorotacrine moiety is crucial to ensure a good hBChE inhibitory activity. Indeed, hybrids retaining this pharmacophore (compounds **15**–**17**, **34**, and **37**) exhibited good to moderate hBChE inhibition, with IC_50_ values spanning the submicromolar to low micromolar range (Table 1 and Supplementary Figure S1). In contrast, hybrids **27**–**29**, in which the 6-chlorotacrine moiety was replaced by simpler heteroaromatic rings, displayed only weak inhibitory activity (around 20% inhibition at a 10 µM concentration). Interestingly, in contrast to the hAChE data, the two linker modifications were associated with a decrease in the hBChE inhibitory potency. In particular, hybrids **34** and **37** bearing modified linkers (amide or PEG) displayed IC_50_ values in the 2–3 µM range. Overall, the most potent hBChE inhibitors within this family were approximately equipotent to 6-chlorotacrine and only slightly less potent than the lead DP-128, confirming that while BChE inhibition is preserved, the structural modifications primarily benefit anti-AChE potency.

**Figure 3:**
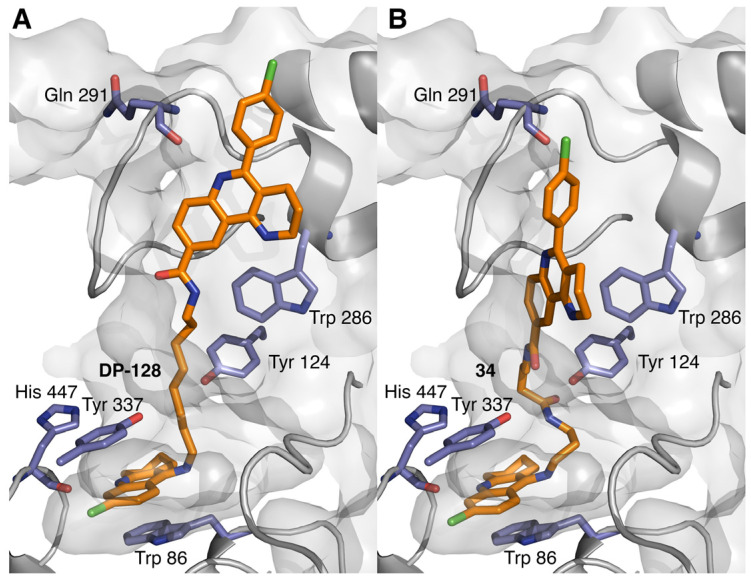
Top-ranked docking solutions of compounds DP-128 and **34** (both coloured in orange) bound to AChE: (**A**) The binding mode of DP-128 displays the traditional interactions established by the 6-chlorotacrine moiety in the CAS and a slightly displaced π-interaction in the PAS, reinforced with an H-bond with the backbone of residue Gln291. The chlorobenzene substituent protrudes from beyond the entrance to the catalytic gorge. (**B**) In addition to similar interactions in the CAS, **34** establishes two hydrogen bonds with residues Tyr124 and Tyr337.

Docking calculations were performed to shed light on the molecular determinants underlying the relatively minor influence of substitution pattern at position 5 of the benzonaphthyridine unit, as well as the distinct behavior observed for compound **34** in AChE and BChE inhibition. To this end, DP-128 and compounds **15**, **16**, and **34** were docked into the crystallographic structures of both enzymes. As expected, in both proteins the 6-chlorotacrine moiety of these compounds occupies the CAS, stacking between the aromatic rings of Trp86 and Tyr337 and establishing a H bond between its protonated quinoline nitrogen and His447, while the linker spans the entire catalytic gorge, positioning the benzonaphthyridine unit between the PAS, where it stacks against the indole ring of Trp286, and the entrance of the enzyme (Figure 3 and Supplementary Figure S2). In the top-ranked binding mode of DP-128 (Figure 3, panel A), the chlorophenyl ring at position 5 of the benzonaphthyridine unit is almost completely solvent-exposed, which is consistent with the low sensitivity of AChE activity to modifications in this ring (compounds **15**–**17**). In the case of compound **34**, the predicted binding mode in AChE is similar; however, the amide group adjacent to the 6-chlorotacrine unit forms two hydrogen bonds with Tyr124 and Tyr337 (Figure 3, panel B). These additional interactions could account for the higher affinity toward AChE of compound **34** compared with DP-128. Notably, both tyrosine residues are absent in BChE, where they are replaced by Gln119 and Ala328, respectively, which cannot form equivalent stabilizing interactions (Supplementary Figure S3). Consistent with this observation, the top-ranked binding mode of compound **34** in BChE does not show any interaction between the amide group and residues in the catalytic gorge (Supplementary Figure S3). The absence of favorable interactions for the amide group likely increases its desolvation penalty upon binding and may therefore explain the different effect that introducing amide linkages has on AChE in comparison with BChE inhibition.

#### 3.3.2. Aβ42 and Tau Antiaggregating Activity

Hybrid compounds designed as dual binding site AChE inhibitors, like DP-128, are typically characterized by the presence of two aromatic or heteroaromatic moieties connected through an appropriate linker. This structural arrangement is intended to promote π-π interactions with key aromatic residues located at both the CAS and PAS of the enzyme. Beyond cholinesterase inhibition, we have consistently found that this class of linked bis(hetero)aryl compounds also displays the ability to inhibit the aggregation of amyloidogenic proteins, like Aβ42 and tau, both in in vitro assays with synthetic peptides and in live *Escherichia coli* cells that overexpress the amyloidogenic protein [51]. Indeed, DP-128 has been previously shown to exert good inhibitory activity against the aggregation of Aβ42 and tau, achieving 74% and 68% inhibition, respectively, at 10 µM in the *E. coli* model [27,28]. Using the same experimental conditions [43], we confirmed that all the newly synthesized DP-128 analogues retained the ability to inhibit the aggregation of Aβ42 and tau. In most cases, the percentages of inhibition exceeded 50% at 10 µM, yielding values broadly comparable to those observed with the parent compound DP-128. Within the series, compound **16** emerged as the most potent antiaggregating agent, with inhibition of Aβ42 aggregation (71% inhibition at 10 µM) comparable to that of the lead DP-128 and inhibition of tau aggregation (80% inhibition at 10 µM) slightly higher (Table 1). This is an interesting result considering that since we developed DP-128, it has been commonly used as a positive reference compound in studies regarding Aβ42 and/or tau aggregation by us and others [70,71,72]. Some of the new analogues, especially **16**, might be likewise used in these studies.

#### 3.3.3. Cytotoxicity of the DP-128 Analogues

The outstanding biological profile of DP-128 prompted us to assess its cytotoxicity. The toxicity of DP-128 was tested in human neuroblastoma SH-SY5Y cells, to find that it was rather toxic (LD_50_ < 10 µM, Table 1), thereby warranting the development of safer analogues, which, indeed, prompted this work. Gratifyingly, 5 out of the 8 novel DP-128 analogues (i.e., **16**, **17**, **27**, **28**, and **34**) turned out to be less cytotoxic than the lead compound, with LD_50_s ranging from 32 to >100 µM (Table 1 and Supplementary Figure S4), in line with our expectations of reduced cytotoxicity upon reduction in lipophilicity. Notably, the 6-chlorotacrine-based derivatives **16**, **17**, and **34** combine potent anticholinesterase and antiaggregating activity, with IC_50_ values in the nanomolar to low micromolar range, and cytotoxic effects observed only at significantly higher concentrations (LD_50_ from 32 to >100 µM). This difference between in vitro potency and cytotoxicity suggests a favorable safety margin for these compounds.

### 3.4. Assessment of DMPK Properties of the DP-128 Analogues: Experimental Determination of Brain Permeability, Aqueous Solubility, and Plasma and Microsomal Stability

The lead DP-128 has high brain permeation [27], as we had determined by the in vitro PAMPA-BBB assay (Table 2). As anticipated, most of the newly synthesized DP-128 analogues, which were designed by the introduction of more polar groups or the removal of lipophilic moieties, showed lower PAMPA-BBB permeability (*Pe*) values than the lead. Nevertheless, their *Pe* values remained clearly above the established threshold for compounds classified as CNS-permeable (CNS+, Table 2), and are, hence, predicted to access their targets within the central nervous system.

When we were getting further insights into the druglike properties of the lead DP-128, we also experimentally determined some physicochemical and pharmacokinetic properties of interest in the context of drug development, namely aqueous solubility, and plasma and microsomal stability in three species (human/mouse/rat). Any compound proposed to be a drug needs some aqueous solubility, as it has to move in the aqueous medium of the organism, to be absorbed, to be distributed to the organ or tissue where its biological target is, and to be metabolized and cleared. The kinetic aqueous solubility of DP-128, determined by nephelometry in DMSO:PBS 1:99 at 37 °C for 2 h, was found to be 14.7 µM (Table 2). Thus, DP-128 is soluble at a concentration that is higher than the concentrations at which it is active on its multiple targets. Half of the new DP-128 analogues turned out to have slightly higher aqueous solubility than the lead, in the range 16.6–20.0 µM, namely compounds **15**, **27**, **28**, and **37**, in agreement with their reduced lipophilicity.

DP-128 and its new analogues contain an amide functionality, which might be sensitive to the action of hydrolytic enzymes that are present in plasma. Satisfactorily, all the tested compounds were found to be quite stable after incubation at 37 °C with human, mouse, and rat plasma. Indeed, all compounds remained essentially unchanged or only barely metabolized after 1 h under the aforementioned conditions (Table 2). Even compound **34**, containing a second amide group within the linker, was found to be very stable, with 99/85/77% of the initial amount remaining unchanged after 1 h.

In contrast, the lead compound DP-128 exhibited limited microsomal stability. Upon incubation at 37 °C with human or mouse liver microsomes, DP-128 underwent extensive metabolic degradation, with only 1.5% and 2.7% of the parent compound remaining after 1 h. A comparatively lower metabolic turnover was observed in rat microsomes, where 36% of the initial compound remained unmetabolized after 1 h of incubation (Table 2). The microsomal liability was apparently a common feature within this structural family. Overall, the newly synthesized DP-128 analogues showed improved stability in rat microsomes, with compound **34** showing the highest stability (43% unchanged after 1 h). However, upon incubation with human microsomes, all analogues underwent nearly complete degradation. A similar trend was observed with mouse microsomes for most derivatives, except for **17**, **28**, and **34**, which remained unchanged to a moderate extent (21–58%) after 1 h incubation with mouse microsomes.

## 4. Conclusions

DP-128 is an anti-Alzheimer multitarget compound, developed in our group some years ago, with a very interesting biological profile. Designed as a dual binding site AChE inhibitor, it is indeed a very potent inhibitor of this enzyme. However, likely its main interest lies in its significant ability to block the aggregation of a large variety of amyloidogenic proteins, including Aβ42 and tau, two prominent actors in AD pathogenesis. In fact, since we developed DP-128, it has been commonly used as a positive reference compound in studies regarding Aβ42 and/or tau aggregation by us and others. While trying to further advance the biological characterization of DP-128, we have found that it suffers from several limitations, such as significant cytotoxicity, moderate aqueous solubility, and poor microsomal stability, which might be ascribed to its rather high lipophilicity.

To overcome these limitations, in this work we have developed a series of DP-128 analogues featuring more polar and/or smaller chemical structures. Half of the new analogues are indeed slightly more soluble and clearly less cytotoxic than the lead DP-128, and retain its high or moderate potency on the multiple targets, hAChE, hBChE, and Aβ42 and tau aggregation, good brain permeation, and high plasma stability. However, the suboptimal microsomal stability seems to be an issue in this structural class, especially human microsomal stability, which should be addressed in further lead optimization endeavours. Meanwhile, some of the new DP-128 analogues, such as hybrids **16** and **17**, have similar or even higher Aβ42 and tau anti-aggregating activity and lower cytotoxicity than the parent compound and might represent novel pharmacological tools in protein aggregation studies.

## Figures and Tables

**Table 1:** In vitro activity of DP-128 and its analogues against their targets (human AChE and human BChE, Aβ42 and tau aggregation) and cytotoxicity in SH-SY5Y cells.

Compound	AChE IC_50_ (nM) ^a^	BChE IC_50_ (nM) ^a^	Aβ42 Aggregation (% inh @10 µM) ^b^	Tau Aggregation (% inh @10 µM) ^b^	SH-SY5Y Toxicity LD_50_ (µM) ^c^
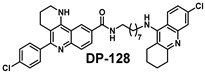	2.06 ± 0.30	286 ± 35	74 ± 4.1	68 ± 3.2	<10
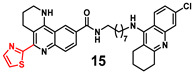	5.05 ± 0.32	1095 ± 85	56 ± 6.0	54 ± 5.2	<10
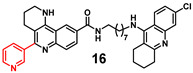	4.51 ± 0.41	415 ± 22	71 ± 2.2	80 ± 1.1	32 ± 18
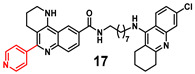	4.89 ± 0.37	504 ± 22	66 ± 3.9	65 ± 2.3	>100
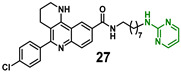	>10,000 ^d^	>10,000 ^e^	51 ± 4.1	66 ± 3.1	76 ± 11
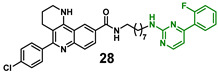	>10,000 ^f^	>10,000 ^g^	42 ± 4.8	61 ± 5.1	98 ± 8.6
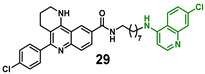	1183 ± 94	>10,000 ^h^	28 ± 5.1	38 ± 3.2	<10
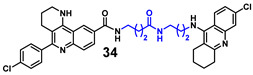	1.31 ± 0.12	2996 ± 209	51 ± 4.1	62 ± 1.7	>100
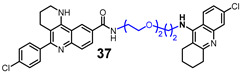	39.4 ± 2.1	2405 ± 173	50 ± 3.1	64 ± 4.3	<10

^a^ Concentration that inhibits by 50% the activity of human AChE or human BChE. IC_50_s are expressed as mean ± SEM of at least 2 experiments, each performed in triplicate. 6-Chlorotacrine was used as a reference compound, IC_50_ hAChE = 14.5 ± 0.9 nM, IC_50_ hBChE = 505 ± 28 nM. ^b^ Percentage of inhibition of Aβ42 or tau aggregation by the compounds at a concentration of 10 µM in live *E. coli* cells that overexpress these proteins. The results are expressed as mean values ± SD of 6 experiments, each performed in duplicate. ^c^ Concentration that causes the death of 50% of SH-SY5Y cells. ^d^ IC_50_ = 40,244 ± 2975 nM. ^e^ 20.5 ± 3.0% inhibition at 10 µM. ^f^ 31.3 ± 4.7% inhibition at 10 µM. ^g^ 19.7 ± 0.8% inhibition at 10 µM. ^h^ 24.7 ± 2.9% inhibition at 10 µM.

**Table 2:** In vitro brain permeability, aqueous solubility, and plasma and microsomal stability of DP-128 and its analogues.

Compound	PAMPA-BBB *Pe* (10^−6^ cm/s^−1^) ^a^	Aqueous Solubility (µM) ^b^	Plasma Stability % Remaining (h/m/r) After 1 h ^c^	Microsomal Stability % Remaining (h/m/r) After 1 h ^c^
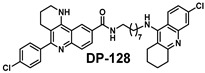	19 ± 1.3 CNS+	14.7	100/97/100	1.5/2.7/36
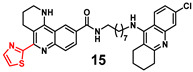	8.6 ± 0.4 CNS+	17.4	100/83/82	1.1/1.4/10
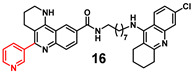	5.8 ± 0.4 CNS+	6.4	100/99/90	2.1/0.9/13
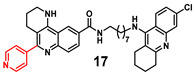	3.5 ± 0.2 CNS±	4.3	100/94/91	1.1/38/3.7
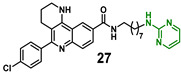	12 ± 1.1 CNS+	20.0	100/79/100	0.3/0.3/5.0
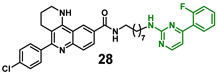	2.2 ± 0.2 CNS±	16.6	100/79/100	1.4/21/2.2
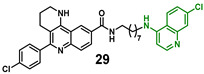	24 ± 1.6 CNS+	13.1	73/100/88	2.3/9.0/17
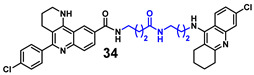	6.1 ± 0.1 CNS+	8.1	99/85/77	1.0/58/43
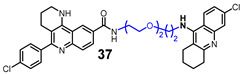	8.4 ± 0.5 CNS+	19.9	100/96/89	0.7/13/16

^a^ PAMPA-BBB experimentally determined brain permeability (*Pe*), expressed as mean ± SD of 3 experiments, each performed in triplicate. The *Pe* values of the reference compounds used in assay validation are shown in Table A1 (Appendix A.1). CNS+ indicates high brain permeability, i.e., *Pe* (10^−6^ cm s^−1^) > 5.1, while CNS± indicates uncertain brain permeability, i.e., 5.1 > *Pe* (10^−6^ cm s^−1^) > 2.0. None of the compounds has low brain permeability (CNS-), i.e., *Pe* (10^−6^ cm s^−1^) < 2.0. ^b^ Kinetic solubility experimentally determined in PBS containing 1% DMSO at 37 °C. Digoxin (solubility = 8.2 µM (described 3.7 µM [45])) and progesterone (solubility = 75.8 µM (described 59.3 µM [45])) were used as reference compounds. ^c^ Percentage of compound that remained unchanged after 1 h incubation at 37 °C with h/m/r (human/mouse/rat) plasma or with h/m/r microsomes.

## Data Availability

The original contributions presented in this study are included in the article/Supplementary Materials. Further inquiries can be directed to the corresponding author.

## Supplementary Materials

The following supporting information can be downloaded at https://www.mdpi.com/article/10.3390/biom16040593/s1. Figure S1: Inhibition curves used for IC_50_ determination against human AChE and human BuChE; Figure S2: Top-ranked docking solutions of compounds **15** and **16** bound to AChE; Figure S3: Superimposition of the top-ranked docking solutions of compound **34** in AChE and BChE; Figure S4: Inhibition curves used for LD_50_ determination in SH-SY5Y cells.

## Abbreviations

The following abbreviations are used in this manuscript:

Aβ	β-Amyloid peptide
ACh	Acetylcholine
AChE	Acetylcholinesterase
ACN	Acetonitrile
AD	Alzheimer’s disease
BBB	Blood–brain barrier
BChE	Butyrylcholinesterase
CAS	Catalytic aromatic site
CNS	Central nervous system
CSF	Cerebrospinal fluid
DCM	Dichloromethane
DDQ	2,3-Dichloro-5,6-dicyano-1,4-benzoquinone
DIPEA	Diisopropylethylamine
DMF	Dimethylformamide
DMPK	Drug metabolism and pharmacokinetics
DTNB	5,5′-Dithiobis(2-nitrobenzoic acid)
HATU	Hexafluorophosphate azabenzotriazole tetramethyl uronium
HRMS	High-resolution mass spectrometry
IPTG	Isopropyl 1-thio-β-D-galactopyranoside
PAMPA-BBB	Parallel artificial membrane permeability assay for blood–brain barrier
PAS	Peripheral aromatic site
PEG	Polyethylene glycol
Th-S	Thioflavin-S

## Appendix A

### Appendix A.1

**Table A1:** Experimental and reported PAMPA-BBB permeability (*Pe* 10^−6^ cm s^−1^) of reference drugs.

Drug	Reported *Pe* [43]	Experimental *Pe* ^a^
Cimetidine	0.0	0.7 ± 0.03
Norfloxacin	0.1	0.8 ± 0.05
Ofloxacin	0.8	1.1 ± 0.02
Lomefloxacin	1.1	0.8 ± 0.02
Hydrocortisone	1.9	1.4 ± 0.05
Piroxicam	2.5	2.4 ± 0.07
Clonidine	5.3	6.5 ± 0.05
Corticosterone	5.1	6.7 ± 0.10
Promazine	8.8	13.8 ± 0.30
Progesterone	9.3	16.8 ± 0.30
Desipramine	12.0	17.8 ± 0.10
Imipramine	13.0	12.5 ± 0.20

^a^ Results are expressed as the mean ± SD of three experiments, each performed in triplicate.

### Appendix A.2

**Table A2:** Difference in calculated logP, TPSA and molecular weight of the new DP-128 analogues relative to DP-128.

Compd	Δ logP		Δ TPSA		Δ MW
	Molinspiration	SwissADME	Molinspiration	SwissADME	
**15**	−0.61	−1.17	+12.89	+41.13	−27.42
**16**	−0.67	−1.25	+12.89	+12.89	−33.45
**17**	−0.80	−1.25	+12.89	+12.89	−33.45
**27**	−2.38	−2.84	+12.89	+12.89	−137.60
**28**	−0.64	−1.26	+12.89	+12.89	−43.52
**29**	−0.50	−0.99	+0	+0	−54.09
**34**	−1.34	−1.63	+29.09	+29.10	+14.98
**37**	−1.48	−1.85	+18.46	+18.46	+3.95
