# Supplementary material for: Synthesis and Biological Profiling of New 1,2,3,4-Tetrahydrobenzo[h]naphthyridine-Based Hybrids as Dual Inhibitors of β-Amyloid and Tau Aggregation with Anticholinesterase Activity
Source: Biomolecules. 2026 Apr 16;16(4):593. doi: 10.3390/biom16040593 (PMC13113287; doi:10.3390/biom16040593)
Supplement: Supplementary file 1 [file biomolecules-16-00593-s001.zip › biomolecules-4207299-supplementary.pdf]

Supplementary Material

Synthesis and biological profiling of new 1,2,3,4-tetrahydrobenzo[*h*]naphthyridine-based hybrids as dual inhibitors of β-amyloid and tau aggregation with anticholinesterase activity

Aldrick B. Verano [1,2,†], Anna Sampietro [1,2,†], Ana Mallo-Abreu [1,2], Rosaria Spagnuolo [3], Belén Pérez [4], Manuela Bartolini [3], M. Isabel Loza [5], José Brea [5], Jordi Juárez-Jiménez [6,7], Raimon Sabate [2,6], Carles Galdeano [2,6] and Diego Muñoz-Torrero [1,2,*]

[1] Laboratory of Medicinal Chemistry, Faculty of Pharmacy and Food Sciences, University of Barcelona, Av. Joan XXIII 27-31, E-08028 Barcelona, Spain; aldrickverano@gmail.com, annasampietro@ub.edu, ana.mallo.abreu@ub.edu

[2] Institute of Biomedicine of the University of Barcelona (IBUB), Av. Diagonal 643, E-08028 Barcelona, Spain

[3] Department of Pharmacy and Biotechnology, University of Bologna, Via Belmeloro 6, I-40126 Bologna, Italy; rosaria.spagnuolo@unibo.it, manuela.bartolini3@unibo.it

[4] Department of Pharmacology, Therapeutics and Toxicology, Autonomous University of Barcelona, E-08193 Bellaterra, Spain; belen.perez@uab.cat

[5] BioFarma Research Group, Centro Singular de Investigación en Medicina Molecular y Enfermedades Crónicas (CIMUS), Departamento de Farmacología, Farmacia y Tecnología Farmacéutica, Universidade de Santiago de Compostela, Av. de Barcelona s/n, E-15782, Santiago de Compostela, Spain; mabel.loza@usc.es, pepo.brea@usc.es

[6] Department of Pharmacy and Pharmaceutical Technology and Physical-Chemistry, Faculty of Pharmacy and Food Sciences, University of Barcelona, Av. Joan XXIII 27-31, E-08028 Barcelona, Spain; jordi.juarez@ub.edu, rsabate@ub.edu, cgaldeano@ub.edu

[7] Institut de Química Teòrica i Computacional (IQTC), Facultat de Química i Física, Universitat de Barcelona, C. Martí i Franqués 1, E-08028 Barcelona, Spain

† These Authors contributed equally to this work

* Correspondence: dmunoztorrero@ub.edu

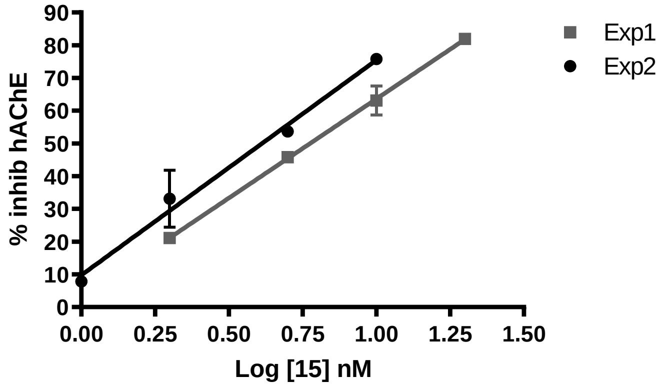
% inhib hAChE
0
10
20
30
40
50
60
70
80
90
0.00
0.25
0.50
0.75
1.00
1.25
1.50
Log [15] nM
Exp1
Exp2

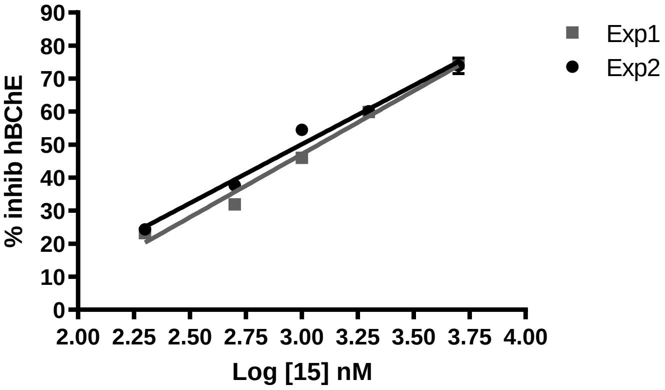
% inhib hBChE
0
10
20
30
40
50
60
70
80
90
2.00
2.25
2.50
2.75
3.00
3.25
3.50
3.75
4.00
Log [15] nM
Exp1
Exp2

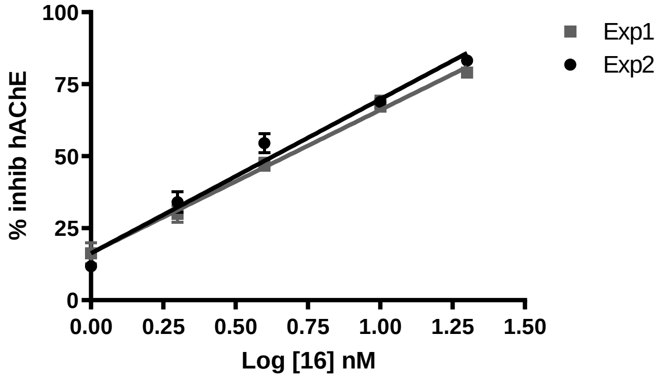
% inhib hAChE
0
25
50
75
100
0.00
0.25
0.50
0.75
1.00
1.25
1.50
Log [16] nM
Exp1
Exp2

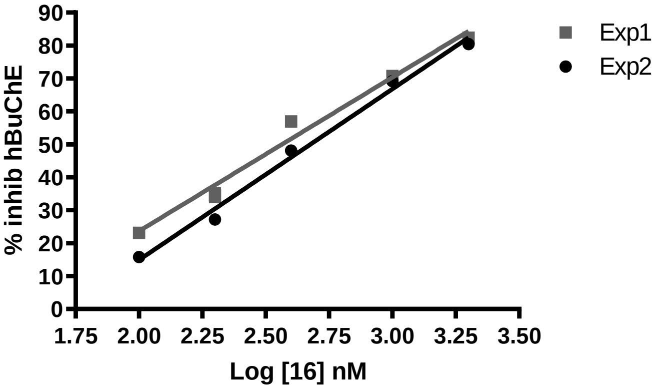
% inhib hBuChE
0
10
20
30
40
50
60
70
80
90
1.75
2.00
2.25
2.50
2.75
3.00
3.25
3.50
Log [16] nM
Exp1
Exp2

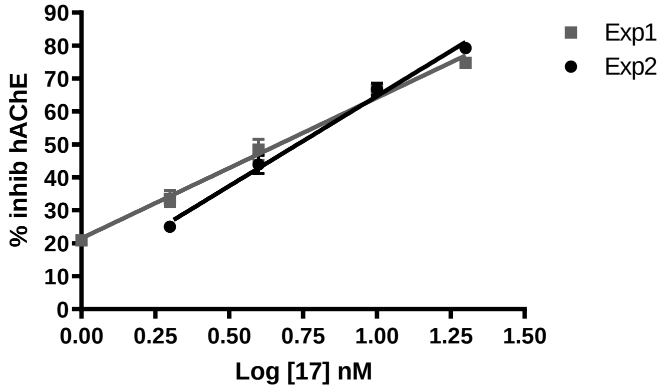
% inhib hAChE
0
10
20
30
40
50
60
70
80
90
0.00
0.25
0.50
0.75
1.00
1.25
1.50
Log [17] nM
Exp1
Exp2

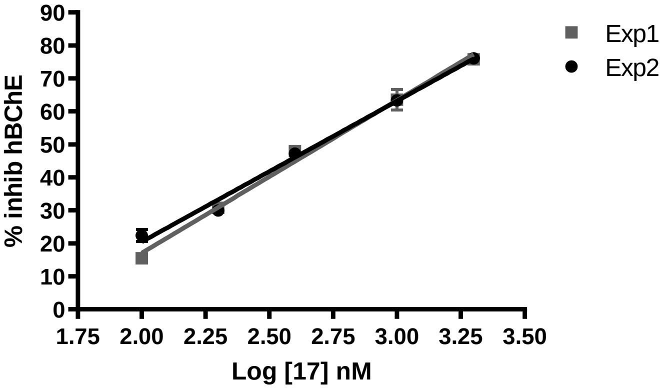
% inhib hBChE
0
10
20
30
40
50
60
70
80
90
1.75
2.00
2.25
2.50
2.75
3.00
3.25
3.50
Log [17] nM
Exp1
Exp2

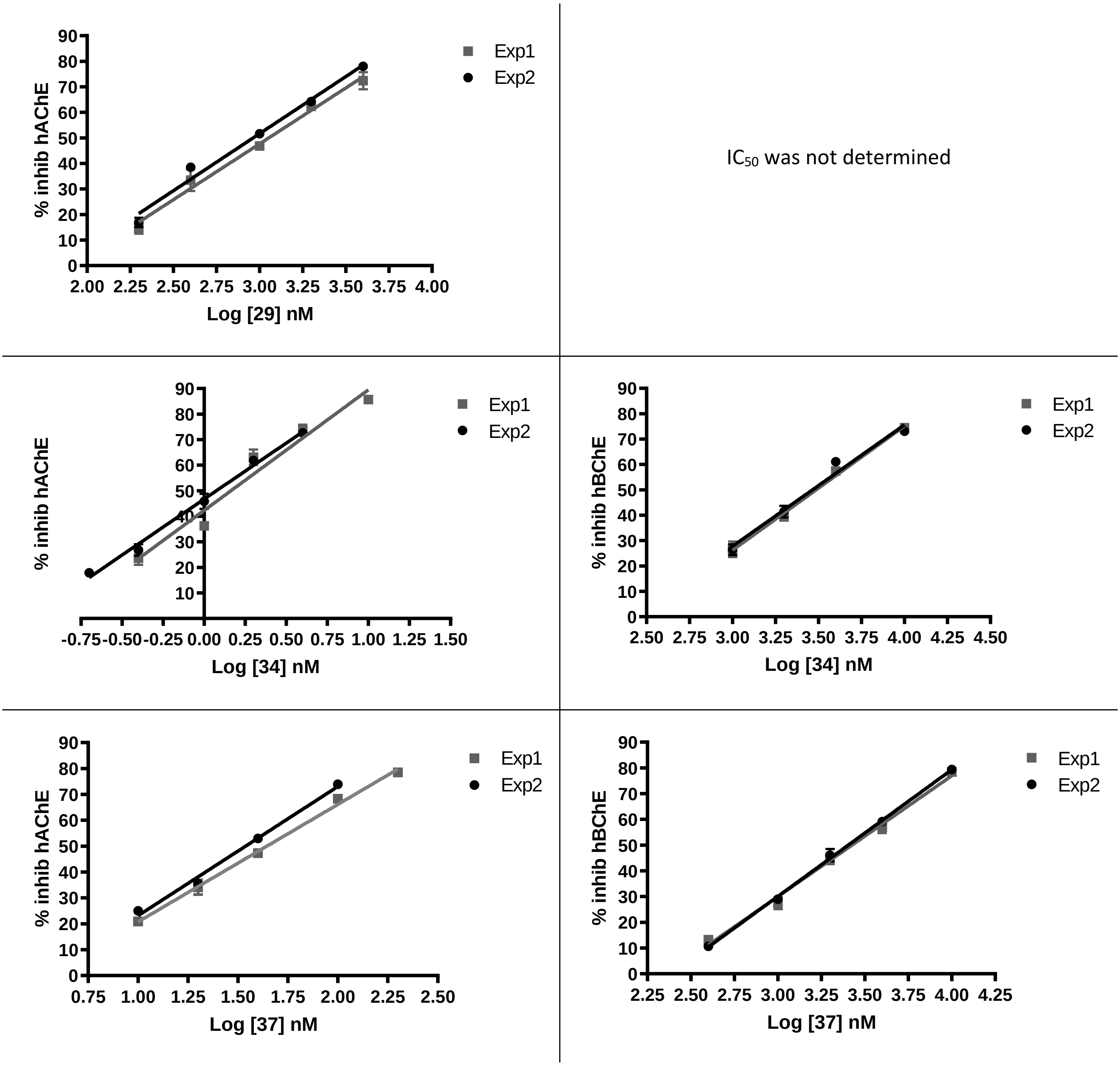

Figure S1. Inhibition curves for the novel 1,2,3,4-tetrahydrobenzo[*h*]naphthyridine-based hybrids used for IC_{50} determination against human recombinant AChE and human serum BuChE. Each compound was assessed in two independent experiments, and data are presented as mean values at each tested concentration with the corresponding error bars. IC_{50} values were calculated using data within the 20–80% inhibition range, corresponding to the most informative and quasi-linear region of the semi-log dose–response curve, thereby minimizing variability associated with the upper and lower plateaus.

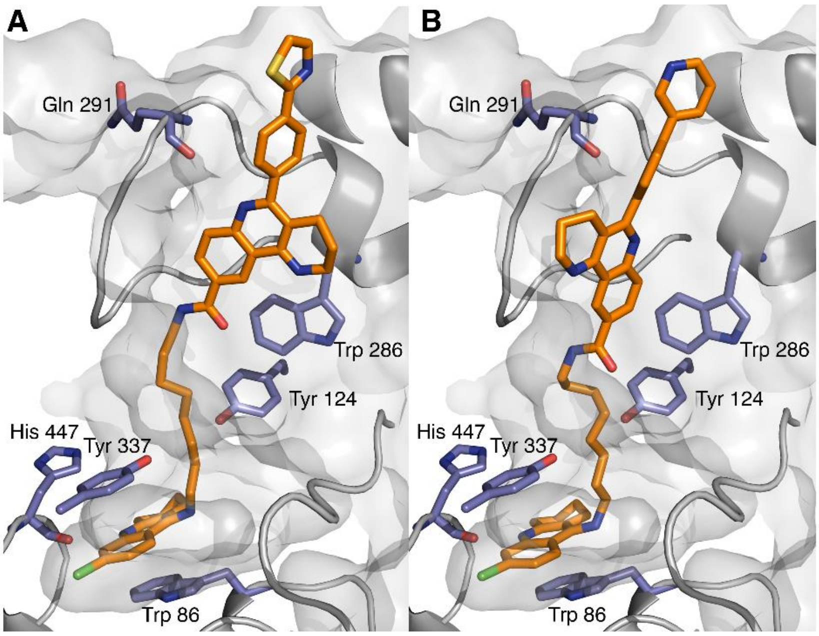

Figure S2. Top-ranked docking solutions of compounds **15** and **16** bound to AChE. A) The binding mode of **15** is similar to the binding mode of DP-128; B) While **16** retains a pattern of interactions for the 6-chlorotacrine unit in the CAS, the benzonaphthyridine unit is rotated by approximately 90 degrees with respect to DP-128.

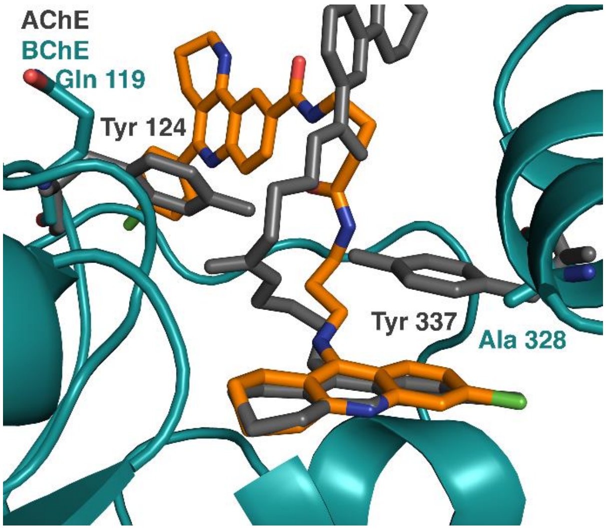

Figure S3. Superimposition of the top-ranked docking solutions of compound **34** in AChE (grey) and BChE (C atoms in teal). The replacement of AChE residues Tyr337 and Tyr124 by Gln119 and Ala328 in BChE is shown in sticks.

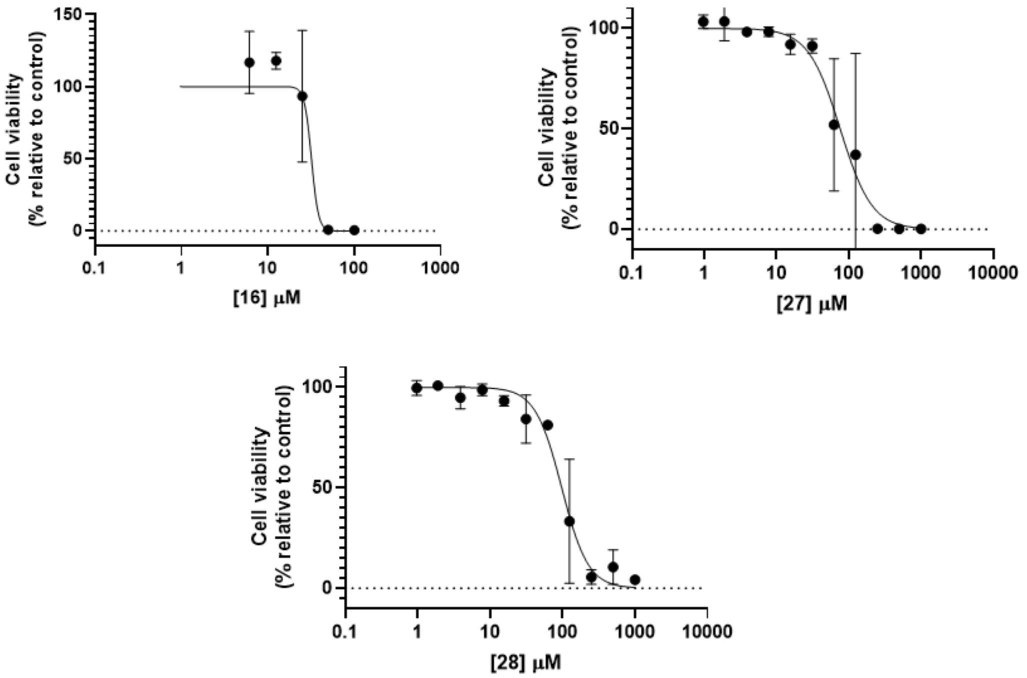

Figure S4. Cell viability curves for the novel 1,2,3,4-tetrahydrobenzo[*h*]naphthyridine-based hybrids used for LD_{50} determination in SH-SY5Y cells.
